# Luminal breast cancer-specific circular RNAs uncovered by a novel tool for data analysis

**DOI:** 10.18632/oncotarget.24522

**Published:** 2018-02-19

**Authors:** Lucia Coscujuela Tarrero, Giulio Ferrero, Valentina Miano, Carlo De Intinis, Laura Ricci, Maddalena Arigoni, Federica Riccardo, Laura Annaratone, Isabella Castellano, Raffaele A. Calogero, Marco Beccuti, Francesca Cordero, Michele De Bortoli

**Affiliations:** ^1^ Center for Molecular Systems Biology, University of Turin, Turin, Italy; ^2^ Department of Clinical and Biological Sciences, University of Turin, Turin, Italy; ^3^ Department of Computer Science, University of Turin, Turin, Italy; ^4^ Department of Molecular Biotechnology and Health Sciences, University of Turin, Turin, Italy; ^5^ Department of Medical Sciences, University of Turin, Turin, Italy

**Keywords:** circRNA, breast cancer, biomarker, alternative splicing, estrogen receptor

## Abstract

Circular RNAs are highly stable molecules present in all eukaryotes generated by distinct transcript processing. We have exploited poly(A-) RNA-Seq data generated in our lab in MCF-7 breast cancer cells to define a compilation of exonic circRNAs more comprehensive than previously existing lists. Development of a novel computational tool, named *CircHunter*, allowed us to more accurately characterize circRNAs and to quantitatively evaluate their expression in publicly available RNA-Seq data from breast cancer cell lines and tumor tissues. We observed and confirmed, by ChIP analysis, that exons involved in circularization events display significantly higher levels of the histone post-transcriptional modification H3K36me3 than non-circularizing exons. This result has potential impact on circRNA biogenesis since H3K36me3 has been involved in alternative splicing mechanisms. By analyzing an Ago-HITS-CLIP dataset we also found that circularizing exons overlapped with an unexpectedly higher number of Ago binding sites than non-circularizing exons. Finally, we observed that a subset of MCF-7 circRNAs are specific to tumor versus normal tissue, while others can distinguish Luminal from other tumor subtypes, thus suggesting that circRNAs can be exploited as novel biomarkers and drug targets for breast cancer.

## INTRODUCTION

Circular RNAs (circRNAs) are transcript isoforms arising from a particular version of alternative splicing, where the 3ʹ-end of an exon is spliced back to the 5ʹ-end of a preceding exon (or itself) in the linear primary transcript [1]. This phenomenon is called “Back-Splicing” (BS) and, exactly as other forms of alternative splicing, it may depend on the permanence of unspliced exon borders during co-transcriptional processing. Mechanisms of RNA circularization not involving canonical splice sites also exist and recently a large collection of intronic circRNAs was published [2]. CircRNAs have been described in all Eukaryotes, in all tissues examined, both normal and pathological, including tumors, and in different stages of development, and show marked cell-specificity [3, 4].

Since not all the genes, and not all the exons within a gene, are capable of giving rise to circular forms the question of which feature(s) may distinguish circRNA host from non-host genes is an important issue. Albeit some distinctive traits of host genes were reported, such as for example increased intron length, the enrichment of RNA editing events [5] and the presence of inverted SINE repeats in introns flanking circularizing exons, leading to possible intron pairing, there is no definitive answer to this question [6]. Some proteins have also been related to biogenesis of circRNAs like ADAR1 [7] or Quaking [8]. As far as their function is concerned, in view of their elevated stability their role as competing endogenous RNA (ceRNA) for miRNA was proposed, at least for some circRNAs [9]. It was demonstrated that circRNA can also function in gene regulation by competing with linear splicing [10]. In addition, more recently several reports testifying translation of ORFs re-created by the BS mechanism were published [11, 12].

The biogenesis of circRNAs is very slow in the cells [13], but since they are extremely resistant to degradation, due to the absence of free ends, the levels of expression of a number of circRNAs in various tissues, like brain, is relatively high [14]. Resistance to exonucleases also makes them long living in the extracellular environment, and many groups are trying to establish the potential value of circRNAs as disease markers in body fluids [15, 16].

One critical issue in seeking information on circRNAs is the usability of the large amount of public data available from normal tissues, cell lines and tumors. Many data were obtained using poly(A)-selected RNA, where circRNAs are almost absent [17]. Instead, poly(A)+ RNA depletion or linear RNA digestion with exonuclease R should be employed as enrichment strategy to detect circRNAs [2]. Total RNA-Seq data can be used to detect circRNAs using the reads overlapping BS junctions, even though the reads mapping to BS junctions are a tiny fraction of total reads commonly detected in RNA-seq experiments.

These limitations apply also to Breast Cancer (BC), where despite the high number of cases in databases, very few of them have the features required to detect circRNAs. Studies have been published reporting pilot analysis of few samples [18] or directly addressing circRNAs in body fluids / exosomes [19]. One study reported circRNAs expression specific to BC subtypes, but since the source was The Cancer Genome Atlas (TCGA) data, which were obtained mainly from poly(A)+ preparations, the number of circRNAs detected in this study was very low [20]. A low number of differentially expressed circRNAs was also reported comparing tumors to adjacent tissues [21].

In BC, despite extensive molecular characterization [22], both novel potential drug targets and circulating biomarkers are eagerly awaited. This is especially true in the case of the so-called “triple-negative” subtype, where no specific driver alteration has been identified so far and, consequently, no specific treatment is available. However, also in the case of the generally less aggressive “luminal” subtype, which is approached with endocrine treatments quite successfully, non-invasive markers able to predict the occurrence of pharmacological resistance may significantly reduce the risk of relapse.

In this work, we describe a large set of circRNAs expressed in MCF-7 BC cells and present an extensive characterization and integration of data available from other cell lines, as well as breast tumor tissues, thanks to the implementation of a novel algorithm to detect and quantitate rigorously BS junctions. Our results illustrate on one side novel findings potentially important to understand the biogenesis mechanism and possible functions of circRNAs. On the other side, several circRNAs expressed in tumor tissues and cell lines display features of biomarkers distinguishing the different subtypes of BC.

## RESULTS

### A comprehensive prediction of circRNAs expressed in the MCF-7 BC cell line

**Figure 1:**
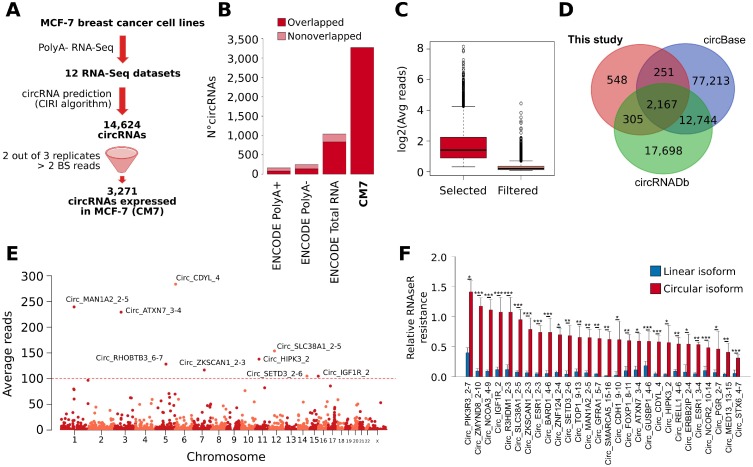
(**A**) Schematic representation of the computational pipeline applied for our prediction of CircRNAs expressed in MCF-7 cell lines (CM7). BS = back-splicing. (**B**) Bar plot represents the number of circRNAs predicted in ENCODE MCF-7 Poly(A)+, Poly(A)-, Total RNA-seq, and in this study (CM7). The dark red color represents the fraction of circRNAs predicted belonging to the CM7 set. (**C**) Box plot showing the log2 average number of BS reads supporting the circRNAs selected (red) or filtered (pink) in our analysis. (**D**) Venn diagram shows the overlap between circRNAs predicted in this study and the annotations from circBase and circRNAdb. (**E**) Manhattan plot shows the number of circRNAs predicted on individual human chromosome. The identifiers of circRNAs supported by more than 100 reads are reported. (**F**) Bar plot represents the RNase R resistance of a set of circRNA predicted in our analysis (red) and of the linear transcripts generated from the corresponding host gene (blue). Standard deviations are computed from five independent biological replicates. *p*-value from Student *t* test. ^***^ = *p*-value < 0.001; ^*^= *p*-value < 0.01; ^**^= *p*-value < 0.05.

The MCF-7 BC cell line is the most widely used *in vitro* model system to represent the estrogen-dependent, invasive luminal A breast tumor subtype. To extend the collection of circRNAs expressed in this cell line, we analyzed twelve datasets relative to a high-depth RNA-seq analysis of poly(A-) preparations from MCF-7 cells cultured in four different culture conditions, each in triplicate, obtained in our laboratory (Materials and Methods). The RNA-seq data were analyzed using the CIRI algorithm [23]. Among 14,624 circRNAs predicted by CIRI, 3,271 candidate circRNAs were selected after the application of several filters (Materials and Methods). The final set of 3,271 circRNAs, thereafter indicated as CM7 (Circular RNAs expressed in MCF-7), was subjected to further analysis (Figure 1A). Note that 79.4% of CM7 were predicted also by the CircExplorer [24] or by the find_circ [1] algorithms (Supplementary Figure 1A).

To confirm that this list may be more comprehensive than other available, we run the CIRI algorithm with the same setting as above on re-mapped ENCODE RNA-seq datasets relative to total RNA, poly(A+) and poly(A-) preparations from MCF-7 cells, obtaining 1,119 (74.5% of overlap with respect to CM7), 161 (50.1% of overlap with respect to CM7), and 244 (55.7% of overlap with respect to CM7) predictions, respectively (Figure 1B). Thus, we concluded that our poly(A-) RNA-seq data possess definitely deeper circRNAs detection power.

CM7 BS sequences were supported on average by 4.52 reads, and 289 of them were supported by more than 10 reads (Figure 1C and Supplementary Table 1A). 2,723 circRNAs (83.2%) in our set are annotated in the public circRNA databases circBase [25] and circRNADb [26], confirming that our predictions are widely reproducible (Figure 1D, Supplementary Table 1B). Noteworthy, 548 CM7 circRNAs have not been described previously in breast or other tissues. However, it should be noted that these predictions were supported on average by a low number of reads (1.28) (Supplementary Table 1B). Some novel circRNAs were confirmed also by Sanger sequencing (Supplementary Data 1).

To accurately characterize circRNAs we developed a novel tool, namely *CircHunter,* allowing accurate annotation, characterization, and expression quantification of a circRNA set, given a reference set of transcripts (see Materials and Methods for details). The *CircHunter*-based annotation of our circRNA set revealed a primary involvement of multi-exonic BS (2,614 circRNAs, 79.9%) with minor number of intronic (163 circRNAs, 4.9%) and intergenic BS events (184 circRNAs, 5.6%) (Supplementary Figure 1B). We reported also special cases, including mono-exonic circRNAs (178 circRNAs, 5.4%), and circRNAs in which BS does not involve exon boundaries but is predicted within exons (134 circRNAs, 4.1%).

Given the extensive heterogeneity of circRNA annotations, we defined a nomenclature for circRNAs. This nomenclature reports the name of the genes involved in the circularization (thereafter indicated as “host genes”) and the rank of exons involved, for each circRNA. Then, considering the average number of BS reads as a proxy of the level of circRNA expression, we identified *Circ_CDYL_4* (283.64 reads), *Circ_MAN1A2_2-5* (239.45 reads), *Circ_ATXN7_3-4* (229.25 reads), *Circ_SLC38A1_2-5* (153.85 reads), and *Circ_HIPK3_2* (137.81 reads) as the most abundant circRNAs in our set (Figure 1E and Supplementary Table 1B).

To experimentally validate CM7, we selected 30 circRNAs covering a range of expression levels reflecting the reads distribution of CM7, and run qRT-PCR analysis using divergent primers designed around the BS junction. The corresponding normal linear mRNA was measured using the forward primer within one exon involved in BS and the reverse primer in the closest exon not involved in circularization. Treatment of RNA preps using RNase R confirmed resistance to exonuclease in 28 circRNAs. As reported in Figure 1F, these circRNAs showed different degree of RNase R resistance, with *Circ_ZMYND8_2-10* and *Circ_NCOA3_4-9* characterized by the lower susceptibility to RNAse R degradation. The two cases that were completely degraded by RNase R were intronic circRNAs and are not shown in the Figure since in this case no linear control was available. Expression levels of the 28 validated circRNAs measured by qRT-PCR in MCF-7 were highly correlated with the expression levels measured by our RNA-seq experiments (Pearson *r* = 0.7611). To verify circRNAs localization in the cell, we performed a cell fractionation experiment. 26 out of the 28 circRNAs tested were enriched in the cytoplasm and only two circRNAs (*Circ_ZNF124_4-2* and *Circ_ZMYND8_2-10*) in the nuclear fraction (Supplementary Figure 1C). These results are consistent with previously published evidence on circRNAs being prevalently found in the cytoplasm [27].

### Genomic circRNAs characterization

**Figure 2:**
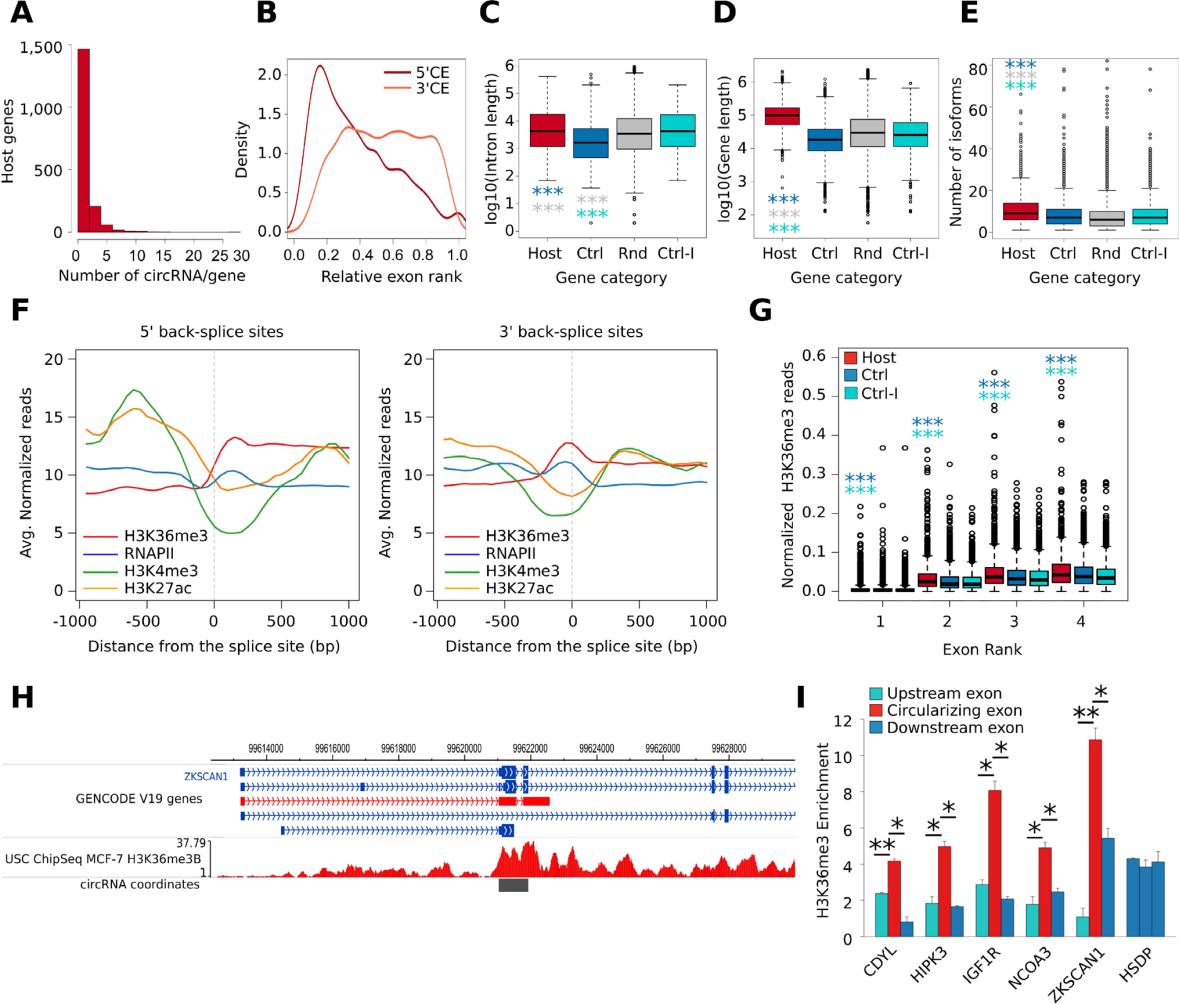
(**A**) Histogram shows the distribution of the number of circRNAs produced by the circRNA host gene identified in our analysis. (**B**) Density plot shows the relative rank of exons involved in circularization. Dark red color represents the 5ʹ Circularizing Exon (5ʹCE) while light red the 3ʹ Circularizing Exon (3ʹCE). (**C**) Box plot shows the gene length distribution of host genes (red), control genes (blue), and random set genes (grey); *p*-value by Wilcoxon Rank-Sum test. ^***^ = *p*-value < 0.001. (**D**) Box plot shows the number of isoforms of host genes (red), control genes (Ctrl, blue), random genes (Rnd, grey), and control genes paired with host genes by the first intron length (Ctrl-I, cyan); *p*-value by Wilcoxon Rank-Sum test. ^***^ = *p*-value < 0.001. (**E**) Box plot shows the length of the first intron of host genes (red), control genes (blue), and random set genes (grey); *p*-value by Wilcoxon Rank-Sum test. ^***^ = *p*-value < 0.001. (**F**) Line plot represents the MCF-7 H3K36me3, Pol II, H3K4me3, and H3K27ac ChIP-seq signal profile measured in a genomic window of +/− 1 kb centered the 5ʹ BS junction (left) and the 3ʹ BS junction (right). (**G**) Box plot shows the number of normalized H3K36me3 ChIP-Seq reads counted in the first four exons of host (red) and control genes (blue); *p*-value by Wilcoxon Rank-Sum test. ^***^ = *p*-value < 0.001. (**H**) Genome Browser representation of the genomic regions involved in the formation of Circ_ZKSCAN1_2-3 circRNA. The H3K36me3 genomic coverage is reported in red. The coverage values are reported as read per million sequenced reads. (**I**) Bar plot represents H3K36me3 ChIP enrichment at the selected exons of five circRNA host genes. In red, the exon involved in the circularization is represented while the flanking exons are represented in blue. The *HSDP* gene was used as representative control gene since it was characterized by a similar level of expression in MCF-7 compared to the five analyzed circRNA host genes. The exons two, three, and four were selected for this gene. Bars indicate standard deviation from three independent biological replicates; ^**^ = *p*-value < 0.01 and ^*^ = *p*-value < 0.05.

We examined the general genomic features of circRNA host genes. As reported in Figure 2A, most host genes generate only one circRNA, even though we observed extreme cases as the *TRIM37* gene, which generates 27 different circRNAs. Considering each circRNA-host gene pair, we did not observe a correlation between their normalized expression level (*r* = 0.05). The capacity of generating circRNAs did not appear randomly distributed, since gene ontology analysis of the circRNA host genes showed significant enrichment of terms related to chromatin modification, DNA repair and cell-cycle, while a set of control genes with no evidence of circular RNA forms (control gene set) was enriched in more general processes including gene expression and translation (Supplementary Table 2A–2B, respectively).

Considering the main transcript isoform of each host gene, we investigated their genomic and transcriptomic features using *CircHunter*. Considering the relative exon rank, we observed that the upstream exon involved in circularization (5ʹ Circularizing Exon, 5ʹCE) is more frequently at the beginning of the transcript, whereas the downstream exon (3ʹ Circularizing Exon, 3ʹCE) has a more uniform distribution (Figure 2B). We observed that the majority of multi-exonic circRNAs involve two (635 circRNAs, 19.4%) or three exons (637 circRNAs, 19.5%) (Supplementary Figure 1D). Exons taking part in the BS events are more frequently the 2^nd^ and 3^rd^ (1,073 and 624 circRNAs respectively) (Supplementary Figure 1E), as previously reported [28]. Also in this analysis, we observed exceptions like *Circ_DYNC1H1_17-56*, in which the circularization event involved 40 exons. Furthermore, intronic regions can be retained in the circRNA structure. To verify intron retention in the circRNAs, we mapped the corresponding paired-end read of each BS-overlapping read in our RNA-Seq. Interestingly, 477 out of 3,164 circRNAs for which it was possible to map the paired read contained intronic sequences (Supplementary Figure 1F), thus demonstrating incomplete splicing of the intervening exons in a number of cases.

To the purpose of identifying further genomic features of CM7, we then collected three sets of control genes: the first (named *control set)* followed these criteria: i) no BS events detected in our RNA-seq nor annotated in circBase; and ii) similar level of expression when compared to the linear isoforms in MCF-7 cells (Supplementary Figure 1G). The second was a subset of *control set* displaying intron length similar to CM7 host genes (named *Control-I* set). Finally, a set of 1,000 random protein-coding genes was selected (named *Random* set). The comparison among CM7, control and random genes led us to conclude that circRNA host genes were significantly longer (Figure 2D) and annotated to a higher number of isoforms (Figure 2E). Furthermore, host genes were associated with a higher number of exons and longer mature RNA transcripts (Supplementary Figure 1H-1I). Moreover, we confirmed that the first intron is significantly longer in circRNA host genes than in control genes (Figure 2C), as previously reported [4].

It has been observed that BS events can be enhanced by the presence of inverted intronic Alu sequences [6]. We analyzed the Alu annotations mapped in the introns flanking the Exons involved in BS and observed that circRNAs host genes were significantly enriched in Alu elements as compared to controls (Supplementary Figure 1J), with 718 host genes (21.95%) harboring a divergent pair of this elements as compared to only 7.65% of non-host control genes.

Thus, the genomic features of CM7 appear in line with those reported for circRNAs characterized in other cell lines and tissues.

### A specific chromatin signature at circularizing exons

Alternative splicing has been linked to epigenetic histone modifications that may result in tighter nucleosome positioning on exons, leading to relenting RNA Polymerase (RNAPII) (the “exon-bump” hypothesis) [29] and/or to the recruitment of splicing factors in the proximity of alternative exons [30]. Therefore, we overlapped BS exons to a library of 15 chromatin states of the MCF-7 epigenome [31] and discovered that they are mostly characterized by the “*transcribed gene*” state, i.e. high H3K36me3 and RNAPII occupancy (Supplementary Figure 1K). Next, we analyzed the ChIP-Seq signal of H3K27ac, H3K36me3, H3K4me3 and RNAPII in a window of +/− 1 kbp centered on the 5ʹBS site (the 5ʹ-end of 5ʹCE) or the 3ʹBS site (the 3ʹ-end of 3ʹCE), using public MCF-7 ChIP-Seq data. Interestingly, we observed a significant increment of the H3K36me3 signal downstream of the 5ʹ BS sites while a local peak of this mark was observed at 3ʹ BS sites (Figure 2F). Then, considering only exonic circRNAs, we compared the increment of H3K36me3 ChIP-Seq signal among the first four exons of host genes and control set, observing that a significantly higher number of H3K36me3 ChIP-Seq reads cover exons of host genes. Interestingly this higher signal was also observed when comparing host genes with the control-I set, suggesting that the enrichment is independent on the intron preceding the 5ʹCE (Figure 2G). To identify circRNA host genes enriched in H3K36me3 signal at 5ʹCEs, we computed a score based on the ratio between the H3K36me3 signal at 5ʹCEs and at their upstream exon. Ranking circRNA host genes using this score revealed interesting examples of genes in which the H3K36me3 signal increases dramatically at 5ʹCEs, as in the case of *ZKSCAN1* (Figure 2H, Supplementary Table 3).

These novel findings are very interesting, especially because the involvement of H3K36me3 in alternative splicing has been reported [30]. To validate our observations by a more direct approach, we used ChIP-qPCR to measure the level of H3K36me3 in five circRNA host genes in MCF-7, by comparing the 5ʹCEs with the upstream and the downstream exons not involved in circularization and to a control gene (HSDP). As shown in Figure 2I, significantly higher levels of H3K36me3 were confirmed in all cases. H3K36me3 enrichment at 5ʹCE is a novel finding that emphasizes the link between alternative splicing and circRNA biogenesis.

### CircHunter tool to search and quantify BS events in CM7

Due to the extension of CM7, we judged it very relevant to see if these circRNAs may represent, or contain, a signature specific to luminal BC. Therefore, we set out to analyze circRNAs expression in external data from BC cell lines and tumor tissue samples. To simplify and speed-up this analysis, we develop *HashCirc*, a module of CircHunter, which applies a two-steps procedure to count the number of reads supporting a putative BS junction.

Briefly, in the first step *HashCirc* decomposes two sets of sequences s_1_ and s_2_ into sub-sequences (*k*-mers), which are then directly compared using a hash-function. In the second step, the s_2_ sequences associated with a threshold of *k*-mers shared with at least one s_1_ sequence are then selected for an alignment performed by the Smith-Waterman algorithm (see Materials and Methods for details). Given the high efficiency of the hash-based sequence comparison, a high number of s_2_ sequences is screened for possible match with the s_1_ sequences to be confirmed afterward using a more accurate (but computationally demanding) alignment method.

To use *HashCirc* starting from the CM7 detected by CIRI we defined a sequence set comprising a window of −35 to +35 nucleotides of each BS. Running *HashCirc* on our 12 RNA-Seq datasets (s_2_) we found that 3,262 circRNAs of our set (99.6%) are associated to at least one read, confirming that *HashCirc* is able to detect almost all CIRI defined circRNAs using a reduced length sequence. Subsequently, *HashCirc* was applied on Poly(A+), Poly(A-), and total RNA-Seq data from the ENCODE project (as described in the first section) showing a neatly higher number of BS reads detected in total RNA-seq data, as compared with experiments based on Poly(A)+ RNA (Supplementary Data 2).

**Figure 3:**
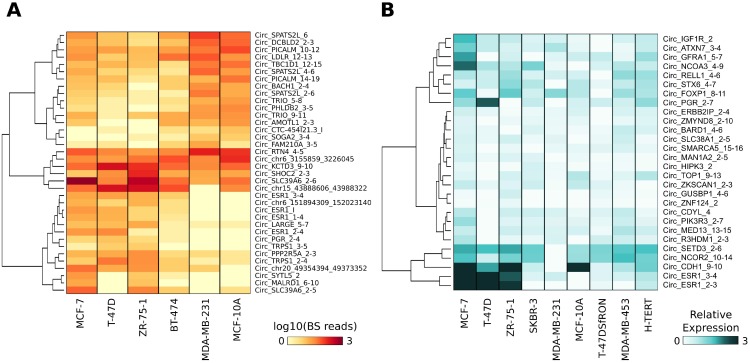
(**A**) Heat map represents the normalized number of BS junction reads of circRNAs differentially expressed in ER+ versus ER- BC cell lines. (**B**) Heat map represents the expression level of a set of 28 validated circRNAs. The expression level was measured by qRT-PCR and is relative to the lowest expressed circRNA in each cell line and is reported in light-to-dark blue color-scale.

We applied *HashCirc* to examine circRNAs expression in publicly available total RNA-seq data of BC cell lines and tissues. Analysis of CM7 BS junction sequences in data from total RNA-seq analysis of five BC cell lines and one non-tumorigenic breast cell line (GSE52643) revealed that 2,037 circRNAs (62.3%) were detectable in at least one cell line; of these, 36 were Differentially Expressed (DE) when comparing ER+ versus ER- cell lines (Figure 3A and Supplementary Table 4). Notably, they included four circRNAs deriving from the *ESR1* gene and one from the *PGR* (progesterone receptor) gene, which are among the genes defining luminal subtypes. To validate this finding, we analyzed by qRT-PCR the expression of the 28 validated circRNAs (Figure 1F) in a panel of cell lines including six BC and two non-tumorigenic breast cell lines, chosen to comprise wide phenotypic variability. We confirmed that *CircRNA_GFRA1_5-7, Circ_IGF1R_2, Circ_ATXN7_3-4* and *Circ_NCOA3_4-9* were prevalently expressed in MCF-7 cells (Figure 3B), in parallel with its linear isoforms (data not shown). Remarkably, a set of circRNAs (*Circ_CDYL_2*, *Circ_MAN1A2_2-5, Circ_SMARCA5_15-16, Circ_FOXP1_8-11,* and *Circ_HIPK3_2*) were more abundant than their corresponding linear isoforms, suggesting an accumulation of these molecules in BC (Supplementary Figure 2A).

**Figure 4:**
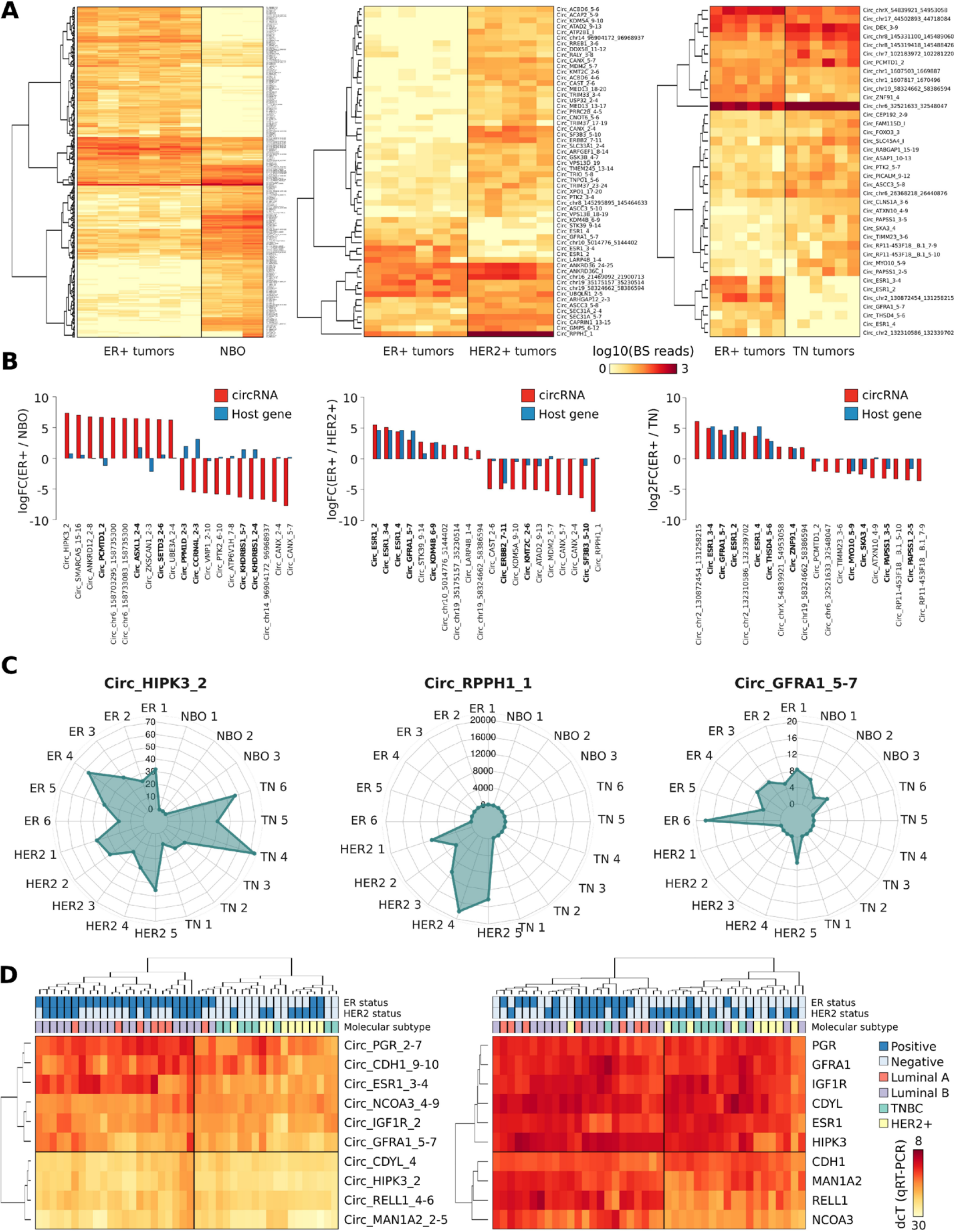
(**A**) Heat map represents the number of BS junction reads of circRNAs significantly Differentially Expressed (DE) in ER+ tumors versus Normal Breast Organoids (NBO) (left), ER+ tumors versus HER2 amplified tumors (middle), or ER+ tumor samples versus Triple Negative (TN) tumors (right). (**B**) Bar plot shows the log2 Fold Change of the ten most significant upregulated or downregulated DE circRNAs in each DE analysis performed (red). In blue are represented their correspondent linear genes. In bold circRNAs whose host genes are known to be DE in a specific condition. (**C**) Radar plot represents the number of BS reads of three representative DE circRNAs, counted by *HashCirc* in 20 total RNA-Seq datasets from primary tumor specimens (ER = ER+ tumors; HER2 = HER2 amplified tumors; NBO = Normal Breast Organoids). (**D**) Heat map represents the expression level of ten selected circRNAs measured by qRT-PCR in 42 different tumor samples, (left) and the level of expression of the corresponding host genes (right). The tumor molecular subtype and the positivity to ER expression or HER2 amplification is reported on top of the heat maps, accordingly to clinical data.

Given the observation of widespread expression of CM7 in BC cell lines, we analysed by *HashCirc* the total RNA-Seq datasets from primary breast tumors and normal tissues [32]. Specifically, we directly searched for circRNA BS junction sequences in 20 RNA-Seq datasets from Triple Negative (TN), ER positive (ER+), HER2 amplified (HER2+), and Normal Breast Organoids (NBO) (GSE52194). We detected 3,004 circRNAs (91.2%) expressed in at least one sample, including 139 circRNAs detected in all the samples analyzed (Supplementary Table 5). To identify luminal-specific circRNAs, we performed a DE analysis using the read counts provided by *HashCirc*. As shown in Figure 4A, we detected 113 DE circRNAs in ER+ *versus* TN tumors, 58 DE circRNAs in ER+ *versus* HER2+ tumors and, noteworthy, 622 DE circRNAs in ER+ *versus* NBO. As expected, the *ESR1* circRNAs were among the most significant DE circRNAs when comparing ER+ to ER- tumors and, similarly, *Circ_ERBB2_7-11* was significantly overexpressed in HER2+ tumors (Supplementary Figure 2B). Interestingly, when comparing luminal tumors against NBO, the differential expression of linear isoforms did not correlate with corresponding DE of circRNAs, while this fact was much less evident in other comparisons between tumor subtypes (Figure 4B). In this analysis, we identified other interesting DE circRNAs including *Circ_HIPK3_3*, which was highly expressed in all tumor samples analyzed; *Circ_GFRA1_5-7*, which was significantly overexpressed in ER+ tumors only; and *Circ_RPPH1_1* as an extremely abundant circRNA in HER2+ tumors (Figure 4C). This circRNA derives from the gene encoding the RNA component of RNase P, and clearly it is not produced by a canonical splicing event since this gene does not contain introns.

Expression of nine circRNAs arbitrarily selected among those showing the highest expression in tumors was also examined by qRT-PCR in a series of 42 tissue samples from primary BC patients. Results were then evaluated by hierarchical clustering and we observed that these circRNAs were able to distinguish samples of the luminal subtype from the other subtypes, with only three misclassified samples (accuracy = 0.92) (Figure 4D, left). Noteworthy, the expression of their corresponding linear isoforms (Figure 4D, right) performed slightly worse as classifier to separate luminal from non-luminal BC subtypes, based on clustering analysis (accuracy = 0.88). Some CM7 resulted thus more efficient in clustering BC subtypes than their linear counterparts, including hormone receptor genes.

Statistical analysis of the expression of these nine circRNAs with patient clinical data highlighted a significant correlation between the immuno-histochemical ER status and *Circ_ESR1_3-4* expression (*p*-value = 3.95E-09), whereas the correlation with ESR1 linear isoforms was definitely less pronounced. The immuno-histochemical PR status was also correlated to *Circ_PGR_2-7* expression (*p*-value = 2.95E-03), as expected. Interestingly, *Circ_IGF1R_2* expression was significantly related to the mitosis score (*p*-value = 4.49E-03) and, furthermore, *Circ_RELL1_4-6* and *Circ_CDH1_9-10* expression was positively correlated with lymph node invasion (*p*-value = 5.64E-03 and *p*-value = 9.30E-03 respectively) (Supplementary Table 6).

### Analysis of Ago-HITS-CLIP data on CM7 exons

**Figure 5:**
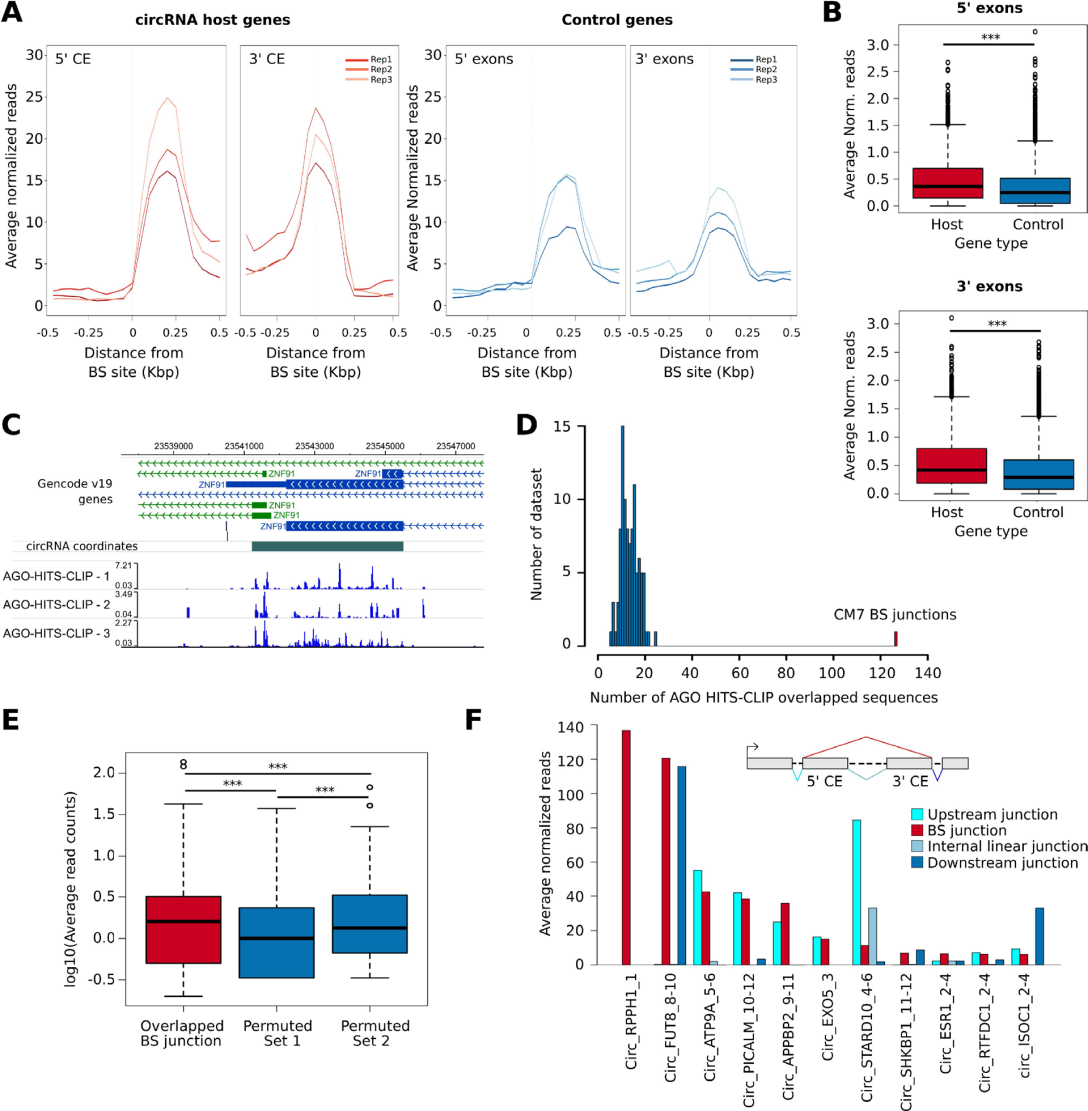
(**A**) Line plot shows the normalized number of Ago-HITS-CLIP reads counted in a genomic window of +/− 500 bp centered on each circRNA BS sites (left) or at corresponding linear splicing sites of genes from the control gene set (right). Data of all the three biological replicates of the experiment are reported. CE, Circularizing exon. (**B**) Box plot represents the number of Ago-HITS-CLIP reads counted within a genomic region of +/− 100 bp centered on each circRNA BS sites (red) or control gene splicing sites (blue). *p*-value by Wilcoxon-Rank Sum Test. ^***^, *p*-value < 0.001. (**C**) Washu Genome Browser representation of the circRNA predicted at ZNF91 gene. The normalized genomic coverage of the three Ago-HITS-CLIP experiments is reported at the bottom. The signal was normalized on the total number of reads sequenced in each experiment. (**D**) Histogram representing the number of sequences overlapped with Ago-HITS-CLIP reads considering the CM7 BS junctions (red) and 100 sets of 3,271 sequences generated by random permutation of CM7 BS sequences halves (blue). (**E**) Box plot representing the log10 average reads in three different datasets: 127 AGO-overlapped back-splicing sequences (red), 100 sets of random sequences generated by randomly permuting 127 randomly selected from the 3,271 CM7 back-splicing sequence halves (Permuted Set 1, blue) and 100 sets of random sequences generated by permuting the 127 back-splicing sequences overlapped with AGO (Permuted Set 2, blue) *p*-value by Wilcoxon-Rank Sum Test. ^***^, *p*-value < 0.001. (**F**) Bar plot showing the average normalized Ago reads overlapped with the top 10 CM7 BS junction (red) and a set composed of linear mRNA splicing sequences in between of the two exons involved in the circularization (light blue). The internal junction was defined by considering the 5’ CE and the following exon in the linear transcript. For Circ_RPPH1_1, mapped on a monoexonic gene, no control junction was available. For the monoexonic circRNA Circ_EXO5_3 no internal junction was available. The number of reads counted on the linear mRNA splicing sequences involving the CE and the flanking exon at the 5’ (cyan) and the flanking exon at the 3’ (blue) were also reported.

The availability of an Ago-HITS-CLIP dataset from a published study in MCF-7 cells [33] prompted us to assess the possible enrichment of Ago binding in CM7 exons, as well as within the BS sequences. First, using Ago-HITS-CLIP data we found that 53% of CM7 exons overlapped an Ago HITS-CLIP peak (Supplementary Table 7A). Then, we computed the intensity of Ago binding around a genomic region of 1 kbp centered on the circRNA exon boundaries, as compared to the boundaries of exons 2 and 3 of control genes. Interestingly, the number of Ago-associated reads is significantly higher around BS exons than in the control set (Figure 5A–5B). These results show that AGO binding is higher in circularizing exon, as exemplified in Figure 5C, even though it is not possible to discriminate how binding concerns linear and circular isoforms.

Circularization events create novel sequences that are not present in the linear isoforms. Therefore, we studied whether some of them could align with Ago-HITS-CLIP sequencing reads. Using the *HashCirc* module of *CircHunter,* we searched for CM7 BS sequences (−35; +35) in the raw Ago-HITS-CLIP data and found 127 overlapping sequences. As control, we generated 100 sets of randomly permuted BS sequences, obtaining definitely lower numbers (*p*-value < 0.001, Figure 5D). Thus, HITS-CLIP reads contain sequences that are exclusive to BS junctions. We also counted the HITS-CLIP reads mapping to BS, and found that they were significantly higher than reads counted on 100 sets of randomly permuted BS sequences or on a set of BS created by shuffling single nucleotides within BS sequences (see Materials and Methods section for details) (Figure 5E). Note that only few CM7 BS sequences were associated with high number of HITS-CLIP reads, e.g. *Circ_RPPH1_1, Circ_FUT8_8-10* and few others (Figure 5F and Supplementary Table 7B).

One important point was to understand whether these “novel” HITS-CLIP reads mapping to BS are due to the re-created BS sequence or to the state of circRNAs per se. In attempt to answer this question, we examined in more detail the circRNAs showing at least 6 HIST-CLIP reads over the BS sequence. Following the scheme shown in Figure 5F we counted the HITS-CLIP reads mapping to the BS junction and to linear splicing junctions (both upstream and downstream the circularization event), and internal to it (when involving at least two exons). Results show that, while the internal junctions are negative in almost all cases, the read counts found at BS junction are reproduced either at the upstream or downstream linear junction (Figure 5F). Mapping of the reads on these junctions, indeed, showed a very asymmetrical distribution, with the peak clearly within the CE (Supplementary Figure 2C).

In conclusion, our data show that there is a clear increase of AGO-HITS-CLIP reads in exons involved in circularization, whereas no evidence of novel HITS-CLIP sequences within the BS junctions was obtained.

## DISCUSSION

In this paper, we present a collection of circRNAs expressed in the MCF-7 BC cell line, which is by far the deepest published to date. We present the new pipeline of analysis *CircHunter*, for the precise circRNAs annotation, and the new tool *HashCirc* (manuscript in preparation) for quantitative analysis of circRNAs expression in public data from tumors and cell lines, showing that a subset of MCF-7 circRNAs can neatly distinguish luminal subtype tumors from other subtypes.

Using both external data and in-house validation, we discovered that circularizing exons contain higher levels of H3K36me3 as compared to other exons, with important implications in the biogenesis mechanism. At the RNA level, we also show elevated Ago occupancy in circularizing exons.

The number of circRNAs detected in our Poly(A-) RNA-Seq was higher compared to circRNAs predicted from ENCODE. Possible explanations are the high sequencing depth of our RNA-Seq data and the fact that samples derived from MCF-7 cells in four different culture conditions, thus enhancing the possibility of detection not only for DE genes, but also from the probabilistic point of view.

During last years, many circRNA prediction algorithms have been developed with different characteristics and performance [34–37]. Among the published algorithms for circRNA prediction, CIRI was selected for its overall good performance and low false negative rate in an independent comparative study of circRNA prediction algorithms [34]. Despite in Hansen and co-workers CIRI was associated with a high false positive rate, we were able to confirm that 79.4% of CIRI predicted circRNA were predicted by at least another algorithm. Furthermore 83, 2% of circRNA were already annotated in circBase. Furthermore, recently in Zhang and co-workers CIRI was associated with the highest precision and sensitivity confirming it as a good balanced algorithm for our porpoise to define a comprehensive set of circRNAs for MCF-7 cells. All the algorithms incorporated a filter on the GT-AG definition of 5ʹ and 3ʹ-splice sites, thus essentially limiting detection to exonic circRNAs. Conversely, a recent study conducted on poly(A)-depleted and RNase R-treated preparations found an impressive number of circRNAs deriving from introns (non-exonic) [2]. Albeit interesting, the undefined and variable junctions in these circRNAs made the evaluation in our dataset unfeasible. It is noteworthy that we found a small number of intronic circRNAs in our set, due to the fact that they featured junctions flanking AG, GT borders by chance. However, we were unable to confirm these non-exonic circRNA by RNase R treatment in two cases selected at random.

The search and quantitation of circRNAs in multiple RNA-seq data is computationally demanding. To improve this task, we developed a pipeline that defines a set of BS sequences and then uses an alignment-free method to compute the matching with RNA-seq data. Alignment-free methods were largely used in genomic data analysis given their inherent low computational cost and their independence from a reference genomic sequence [38]. This method was implemented in the first step of *HashCirc*, making the algorithm faster and well performing as shown for example in the case of circRNAs expressed in breast tumor tissues. CM7 were clearly differentially expressed among tumor subtypes and, even more interestingly, a large number of them were differential in tumors *versus* normal tissue. Even though this conclusion was suggested already by other authors [18–20], to our knowledge, our circRNAs set is the largest reported to date at least for the luminal subtype breast tumors. Nair and co-workers also reported several tumor- and subtype-specific circRNAs in BC. However, this study was conducted using poly(A)+ RNA-seq data, thus strongly limiting the detection of circRNAs, which do not present poly(A)+ tails. The risk of introducing a bias toward circRNAs contaminating poly(A+) RNA preparations to variable degrees, as well as toward circRNAs containing poly(A) stretches by chance, discouraged us from examining data from TCGA or other sources not produced using total or RNase R-resistant RNA preparations. It is clear that more studies and larger patient cohorts are needed to reach a robust conclusion on this point.

In the analysis of clinicopathological data we noticed that circRNAs generated by *ESR1* and *PGR* genes show higher correlation with ER and PgR protein level than their corresponding protein-coding isoforms. The interesting hypothesis that this circRNAs may positively influence the translation or stability of their cognate protein is currently under investigation.

When considering the epigenetic status of genes hosting circRNAs, we observed that exons involved in circularization display an unexpected increase of H3K36me3. While H3K36me3 is generally observed within transcribed gene body [39], enrichment at circularizing *versus* non-circularizing exons is a completely novel finding, to our knowledge. This is particularly interesting when the mechanism of biogenesis is considered. In fact, assuming that circRNAs formation takes place during “normal” co-transcriptional processing, one should admit that the upstream intron-exon border and the downstream exon-intron border involved in circularization should be in unspliced form, i.e. the region involved (exons+introns) is “skipped”, in some sense. Luco and co-workers. reported that the H3K36me3 is read by the chromodomain-containing MRG15 protein, which in turn binds the splicing repressor PTB [30]. It should be extremely interesting to see whether a similar mechanism should take place in the case of circRNAs, i.e. the permanence of one or several unspliced intron-exon borders due to H3K36me3-mediated splicing repressor recruitment.

We have explored evidence of AGO binding not only for its role in post-transcriptional regulation, but also for its reported role in transcription and splicing [40]. Indeed, Ago1 binding to intronic enhancers in association to HP1α and CTCF has been correlated to exon skipping [41, 42], making it possible that Ago1 may associate to circRNAs at this level. While it is clear that BS creates novel sequences and some of them could be found in the AGO-HITS-CLIP raw data library, read distribution over circularizing versus linear junctions demonstrated that peaks are inside exons, thus excluding their dependency on sequences recreated by circularization. Nevertheless, AGO reads are quantitatively more abundant at BS junction than random or permuted sequences: this may result from circularization being linked to specific RBP complexes that are not present in linear isoforms, or to higher stability. The same considering the higher density of AGO-HITS-CLIP reads at circularizing *versus* non-circularizing exons.

As far as the possible involvement of microRNA targeting is concerned, target prediction in circularizing exons unravelled interesting cases that are currently being studied in more detail in our lab. The first is Circ_ZNF91_4, which contains 25 potential MREs for miR-23b-3p and 24 MREs for miR-23a-3p (Supplementary Table 7C). It is worth of note that Circ_ZNF91_4 is up-regulated in ER+ tumors compared to NBO and ER- tumors. The second case is miR-193b-3p, which was predicted targeting a number of highly expressed circRNAs, among which Circ_HIPK3_2, which shows high expression in tumors as compared to normal, and as compared to HIPK3 linear isofoms. Among other predicted targets we noticed Circ_CDYL_4, Circ_SPECC1_4, Circ_PTK2_3-5, Circ_PIK3R1_2, and also Circ_ESR1_2-3. It should be stressed, however, that reports suggesting a role of circRNAs as competing endogenous RNA (ceRNA) have been so far anecdotic and that all these predicted interactions have to be verified in the lab. In this work, we did not address the possible regulation of circRNAs by estrogen. Along with the slow turnover of circRNAs, a serious problem to evaluate regulation consists in the development of robust methods to compare the rates of circRNA biosynthesis and decay with those of their cognate linear isoforms. All these issues are currently being investigated in our lab.

In conclusion, along with data that are simply suggestive on the mechanisms regulating circRNA biogenesis, the data presented here set a solid ground for further studies on the role of circRNAs in tumors. The extended collection of circRNAs expressed in MCF-7 cells and the new tool for external data analysis will enable exploration of circRNAs expression in data from tumor tissues as soon as they become available. Notably, due to the reported stability of circRNAs in serum, the subtype-specific set we report here will represent the basis for development of non-invasive biomarkers in Breast Cancer.

## MATERIALS AND METHODS

### RNA isolation, RNAse enrichment and quantitative Real-time PCR (qRT-PCR)

RNA was isolated from MCF-7, MDA-MB-231, SK-BR-3, T-47D, T-47D-sfRON, ZR-75–1, HTERT-HME1, MDA-MB-453 and MCF-10 cells as reported in [43]. RNase R (Epicentre Biotechnologies) treatment (3U) was performed on total RNA (1μg) at 20°C for 15 min. Cellular RNA fractioning was performed as described in [43]. The qRT-PCR analyses were performed using the SYBR-green method (iTaq Univers SYBR Green, Biorad, 1725124). Real-time PCR primers for human 18S (QT00199367), ERα (QT00044492), GREB1 (QT00080262), GUSBP1 (QT00085204) and RELL1 (QT01662647) RNAs were purchased from Qiagen (QuantiTect@ Primer Assay). Custom expression-primer pairs are reported Supplementary Table 8.

### Sanger sequencing

The PCR products were subjected to electrophoresis in 0.8% agarose gel. The fragments were purified and quantified. The Sanger sequencing of PCR products was performed by Bio-Fab research s.r.l. (Rome, Italy) and reported in Supplemental Data 1.

### Chromatin immunoprecipitation assay (ChIP)

MCF-7 cells were grown in serum enriched medium (full medium) and ChIP experiments were performed as described in [44]. In this assay was used an antibody against H3K36me3 (Active Motif, cat. 61101, lot. 32412003) and Normal Rabbit IgG (Millipore, cat. 12-370). Custom ChIP-primer pairs are reported in Supplementary Table 8.

### Small interfering RNA (siRNA)

MCF-7 cells were reverse transfected with siRNAs (20nM final concentration) using Lipofectamine 2000 (Thermo Fisher Scientific, 11668-019). Stealth RNAi from Thermo Fisher Scientific were used to target ERα mRNA (Thermo Fisher Scientific ESR1HSS103376, ESR1HSS103377, ESR1HSS176619). Stealth RNAi™ siRNA Negative Control Med GC was used as a control (siCTR; Thermo Fisher Scientific, 12935–300). Cells were harvested 48 hours after siRNA transfection.

### Starting RNA-Seq datasets and initial prediction of circRNAs in MCF-7

The starting RNA-seq datasets were obtained from libraries generated with the TruSeq stranded library preparation kit (Illumina) using RNA depleted of both poly(A+) and ribosomal RNAs fractions. Libraries were analyzed with the DNA 1000 chip (Agilent) using Agilent 2100 Bioanalyzer and quantified using the Qubit DNA HS kit (Lifetechnologies). Pool of 12 libraries (pooled at equimolar concentration) was generated, quantified and run on the HiSeq2000 (Illumina) sequencer in 50 nt paired-end sequencing mode following manufacturer instruction. A total of 12 datasets, with an average depth from 30.7 to 116.1 million paired-end reads were obtained, composed of triplicates of four MCF-7 culture conditions: i) hormone-deprived (HD) media ii) HD+ 17β-estradiol (6h) iii) medium added of FBS 10% iv) double-stranded RNA complementary to ESR1 mRNA (siRNA) (48h). Raw data are deposited at GSE101410. CIRI v. 1.2 [23] circRNA prediction analysis was performed aligning RNA-Seq reads with BWA v. 0.6.1 [45] with option *bwasw* and -*T* = 15. Gencode v19 was used as reference transcriptome dataset while hg19 as human reference genome assembly. CIRI algorithm applied in default settings with -*P* and -*low* option.

CircRNA prediction with CIRCexplorer v. 1.0.6 was performed as proposed in [24]. RNA-Seq reads were aligned using Tophat v. 2.0.0 [46] with options -*bowtie1*, -*a* = 6, -*m* = 2 -*microexon-search* -*no-novel-juncs*. Unmapped reads were analyzed with Tophat-Fusion with options -*fusion-search* -*keep-fasta-order* -*bowtie1* -*no-coverage-search.* CircRNA prediction analysis with find_circ v. 1.2 [1] was performed by aligning reads using Bowtie v. 2.0 [47] with options -*very-sensitive* -*phred33* -*mm* -*D* = 20 -*score-min* = C,-15,0. Unmapped reads were used as input for the find_circ pipeline following the procedure proposed in [1]. For each analysis, the number of BS reads reported by each algorithm was normalized using DESeq2 v.1.14.1 R package [48]. On each set of circRNA predicted by the three algorithms, the circRNAs predicted in at least two out of the three biological replicates in each culture condition and associated with an average number of BS supporting read > 2 were selected. Using this threshold 3,271, 1,811, and 2,797 circRNAs were predicted with CIRI, find_circ and CircExplorer, respectively. CIRI algorithm was applied with the same settings to predict circRNA from ENCODE MCF-7 RNA-Seq experiments performed using total RNA (GSM2072571, GSM2072572), poly(A)+ (GSM767851), and poly(A)- (GSM765388) RNA selection protocols. Only circRNAs identified in both the biological replicates of the experiments were considered for the analysis.

### CircHunter tool

*CircHunter* is a new tool designed for the post-discovery analysis of circRNA predictions. CircHunter is composed by three modules (i) circRNA classification, (ii) BS sequence reconstruction, and (iii) BS sequence quantification in deep sequencing datasets.

In the circRNA classification module the algorithm considers the annotation from a reference transcriptome, which in our analysis was Ensembl v85. Initially, genomic coordinates of each Ensembl exon are overlapped against circRNA genomic coordinates using bedtools [49] *intersect* function. Then, the genomic coordinates of each exon annotation are tested for the overlap against circRNA BS site position. Each overlap is classified based on the number and the position of the BS within the transcript annotations. Each circRNA/transcript overlap is classified to five possible criteria (Supplementary Figure 1B):

- *multiexonic*, when two exons are mapped to each splice site of the circRNA;
- *monoexonic,* when a single exon spans the entire region involved in the circularization;
- *putative exonic*, when there is no precise match between the circRNA BS sites and the exon boundaries but BS sites is mapped within exon genomic coordinates;
- *vintronic*; when at least one intron is mapped to a circRNA BS site;
- *intergenic*; when at least one circRNA BS sites exceeds the boundaries of the associated gene.

This analysis provides a transcript-level classification of circRNA-overlapping transcript. Then, a single circRNA can be associated with multiple classifications when overlapped on multiple transcripts. To obtain the univocal classification of each circRNA, the main isoform of the circRNA host genes is considered by selecting the Ensembl transcript identified with the suffix “001”. If none main isoform is overlapped with a circRNA, the other isoforms are evaluated following the order provided by Ensembl.

The circRNA nomenclature applied in this work was based on the isoform considered for the univocal classification. Specifically, each circRNA name was composed by the prefix “Circ” followed by the host gene symbol and ended with the rank of 5ʹ and 3ʹ exons involved in the circularization. Intergenic circRNAs were named based reporting their genomic coordinates while intronic circRNAs were distinct by the “I” suffix. The univocal classification was considered in the analysis of the number and rank of the exons involved in the BS event.

The circRNA BS sequence reconstruction module applied a python script which select two set of genomic coordinates starting from the BS sites and involving a portion of BS exon selected by the user (default length is 35 bp). An R script is then applied to convert the genomic coordinates in R *GRanges* objects. Then, the BS sequence is reconstructed using the function *getSeq* and *xscat*. These functions were applied respectively to extract and to concatenate properly the two sequences composing the BS junction.

The BS sequence quantification in deep sequencing datasets module is performed by *HashCirc*. *HashCirc* is organized on three steps: in the first and second steps an alignment-free prediction method is exploited to identify the set of putative sequencing reads mapped on the sequences of interest; while in the third step the selected putative reads are aligned against the sequences of interest (i.e. circRNA BS junction sequences) to generate the corresponding *counting table* (i.e. the counting of the number of reads aligned with each sequence).

*Step 1: Significant k-mer generation*. In this step, the entire set of sequences is scanned and a set of substrings with length *k*, namely k-mers, is generated using a *sliding window* approach.

For instance, given a string *ATCCCGTC* the following k-mers with length three are generated: *ATC, TCC, CCC, CCG, CGT* and *GTC.*

Then, a hashing is exploited to build the function *isPresent*: {A,C,G,T}_k_ → [0,1] which, for each k-mer, returns one if it appears in any sequence otherwise 0.

A k-mer α is considered significant and therefore selected if *isPresent(*α) = 1. These selected k-mers will be used to identify the putative reads in the next step.

*Step 2: read selection*. In this step hashing is still used to build function *check*:{A,C,G,T}_k_ →{*0,1*}, which for each k-mer returns *1* if it is a selected k-mer otherwise *0*. Then, the function *check* is applied on all the k-mers of a read so that a read is selected as putative one if it contains more than *N* k-mers for which *check* function returns 1.

*Step 3: read counting*. The derived set of putative reads are hence aligned w.r.t the sequences. For each read, its best alignment with respect to all the sequences is identified and used to generate the sequence counting table. In the CircHunter tool suite, the *HashCirc module*, is composed of two C++ applications for each step of the data processing:

The first step takes as input a set of sample reads, the set of sequences and the threshold *N,* and returns the corresponding set of putative reads which contain at least N k-mer shared with the set of sequences. The k-mers generated by the sequences are stored in RAM exploring an ad-hoc C++ hash table class implementation to optimize the trade-off between the memory utilization and the execution time.

The second step takes as input the set of putative reads for each sample, it counts the frequency of a set of reference sequences (i.e. pre-defined BS sequences). For this step, the Smith-Waterman algorithm provided by SIMD Smith-Waterman C++ library is used.

To perform the *HashCirc* analysis, sequences of 70 bp representing the hypothetical circRNA BS junctions were extracted from the circRNA predictions using *CircHunter*. Two set of genomic coordinates spanning +35/−35 bp from the junction point respectively were generated using this algorithm and circRNAs shorter than 70 bp were splitted in two halves used for the junction reconstruction. The efficiency of *HashCirc* in circRNA quantification was evaluated by Pearson correlation analysis between the number of reads counted by *HashCirc* with the reads reported by CIRI. ENCODE MCF-7 RNA-Seq experiments were also considered for analysis of the algorithm sensitivity in circRNA detection. For this analysis data from Poly(A)+ (GSM767851), Poly(A)- (GSM765388), and total RNA-Seq datasets (GSM2072571, GSM2072572) were analyzed by setting the k-mer length (*k*) to 26, the minimum number of matched k-mer (*N*) to 21, and the minimum number of matches (*M*) equal 40. A set of 75-bp simulated paired-end reads from [50] was considered as circRNA negative set since it was generated from linear mRNA annotations. A ratio between the reads counted by HashCirc in the PolyA- and the simulated read datasets was computed to evaluate the rate of false positive count. The results of these testing analyses are reported in Supplementary Data 1.

To make easy the use of this new pipeline a Graphical User Interface (GUI) based on Java Swing Class was developed too. Moreover, the pipeline was integrated into a Docker container to facilitate its distribution and installation. It can be downloaded at https://github.com/carlo-deintinis/circhunter

### Public RNA-Seq analysis

The public RNA-Seq experiments were analyzed by reads alignment against Gencode v19 annotations and Hg19 genome using Tophat v. 2.0.0 in default settings. Read count was performed using the featureCount algorithm v.1.5.0-p1 [51] and read count table normalized using DESeq2 v.1.14.1 R package. Normalized read counts were then converted in Fragment Per Kilobase exon per Million mapped reads (FPKM) considering the length of the longest isoform and the million number of read counted by featureCount.

### circRNAs host genes genomic characterization

The *Control gene set* was defined by selecting genes lacking circRNA predictions considering the union between circBase [25], circRNADb [26] annotations and circRNAs predicted in this study. To select control genes expressed in MCF-7, a public Poly(A)+ MCF-7 RNA-Seq experiment performed in full medium (GSE48213, [52]) was re-analyzed and genes with a FPKM > 1 were considered as expressed. Using these criteria 5,583 control genes were selected. The not significant difference between host and control genes expression was confirmed by Wilcoxon Rank-Sum test (*p*-value = 0.796). Another control set was defined by selecting a subset of control genes paired with circRNA host genes by considering the first intron length. Specifically, a python script was designed to pair each circRNA host gene with a randomly selected control gene with similar intron length. The pairing was performed 1,000 times. This control set was called *Control-I*. The *Random gene set* was composed by 1,000 sets of 1,761 genes randomly selected from Gencode v19 annotations.

Enrichment analysis of circRNA host gene and the Control gene set was performed using Enrichr web tools [53]. The comparison of gene/transcript length, the number of exons and isoforms between circRNA host genes and control set was performed using the Ensembl annotations. Data of the main gene isoforms (reported with suffix “001” by Ensembl) were used in this analysis. Analysis of candidate circRNA intronic retention events was performed by considering reads paired with each BS spanning read. The genomic coordinates of these reads were retrieved from BWA alignment outputs using BS read identifiers provided by CIRI. Then, the reads genomic coordinates were mapped against Ensembl exon genomic coordinates. Only perfectly matched reads were considered for this analysis.

Alu annotations were downloaded from UCSC using the RepeatMasker track and their coordinates were overlapped with an intronic region of 500 bp flanking the circRNA BS junctions as previously performed [6].

### Analysis of the epigenetic status of circRNA genomic regions

The epigenetic status at the genomic region involved in the BS events was evaluated by overlapping the BS genomic coordinates with 15 chromatin states defined for the MCF-7 epigenome in full medium culture condition [31]. Data of ChIP-Seq experiment against H3K27ac (GSM1383859), H3K36me3 (GSM970217), H3K4me3 (GSM1383862) and RNAPII (GSM1276019) were also analyzed to measure the ChIP-Seq genomic signal profile around 5ʹ or 3ʹ BS sites. Specifically, ChIP-Seq reads were aligned against hg19 human genome using Bowtie2 in default settings. The genomic signal profile was then computed using seqMINER algorithm v1.3 [54] considering a genomic region of +/− 1 kbp centered on the BS sites. The genomic signal normalization was then normalized using *NormChIP* algorithm [31]. The number of H3K36me3 read covering the first five exons of circRNA host genes, the control set, or the control-I set were counted using the *coverageBed* function of bedtools.

### Ago-HITS CLIP data analysis and MRE prediction

Raw Ago-HITS-CLIP data and Ago-HITS-CLIP peaks were retrieved from GSE57855. The raw sequencing reads were aligned using Bowtie2 algorithm in default settings and reads aligned within a genomic region of +/− 1 kbp centered on circRNA BS sites were counted. As control the corresponding splicing sites of exon 2 and exon 3 from genes were analyzed.

MRE prediction was performed on the reconstructed sequence of exonic circRNAs composed of one two, or three exons. MRE prediction was performed using the Miranda algorithm [55] in default settings and considering mirBase v20 annotations. Overlap with circRNA exons and Ago-HITS-CLIP was performed using *coverageBed* function of bedtools. To analyze miRNAs expressed in MCF-7 cells, processed data from small RNA-Seq experiments from GSE78168 were considered. Only miRNAs associated with an average Read Per Million Mapped reads (RPPM) > than 100 in E2 untreated cells were considered.

*HashCirc* was applied on Ago-HITS-CLIP to identify Ago-RNA binding generated by BS events. Given the smaller length of Ago-HITS-CLIP reads the algorithm was applied with settings k = 21, N = 17, and M = 30. Read count normalization was performed using DESeq2 algorithm. MRE prediction was performed on the reconstructed BS sequence of circRNA subset associated with an Ago-HITS-CLIP signal as defined by *HashCirc* analysis. Different control sets were defined for this analysis: 1) 100 sets of 3,271 sequences generated by randomly permuting the 3,271 CM7 BS sequences; 2) 100 sets of sequences generated by randomly selecting and permuting 127 CM7 BS sequences; 3) 100 sets generated by randomly permuting the 127 BS sequences overlapped with the Ago-HITS-CLIP datasets; 4) 100 set of sequences generated by randomly shuffling the 3,271 CM7 BS sequences.

Considering the CM7 BS sequences overlapped with more than six averaged read of the Ago-HITS-CLIP, the overlap with the splicing junction of the linear mRNA sequences was evaluated. The overlap was performed by selecting the splicing junction sequence in between of the exons involved in the circularization and also by considering the junction formed between the circularizing exons and the external 5ʹ and 3ʹ flanking exons. Direct sequence alignment between Ago-HITS-CLIP reads and the splicing junctions was performed using Bowtie2 algorithm with *local* option. The read coverage was computed using Samtools *pileup* function.

### CircRNA expression analysis in public total RNA-Seq experiments

The analysis of circRNA expression in five BC cell lines and one non-tumorigenic cell line was performed considering total RNA-Seq experiments from GSE52643. The reads aligned against the circRNA BS junction were counted using *HashCirc* with settings k = 21, N = 17, and M = 30. For the analysis of circRNA expression in primary tumor tissues (GSE52194), total RNA-Seq data of 20 BC samples and 3 NBO were analyzed with *HashCirc* with settings k = 22, N = 18, and M = 33. The different settings were selected based on the different length of the RNA-Seq reads analyzed.

The read count normalization and the differential expression analysis were performed using DESeq2. The analysis on cell lines data was performed between ER+ (MCF-7, T47-D, ZR-75.1) and ER- (BT-474, MDA-MB-231, MCF-10A) cell lines. The analysis of circRNA expression in tumor tissues was performed by comparing ER+ tumors against NBO, HER2+ amplified tumors, or Triple Negative (TN) tumors. A circRNA was considered significantly Differentially Expressed (DE) if associated with a *p*-value < 0.05.

Radar plot representation of candidate circRNAs expression in tumor tissues was performed using the *fmsb* R package.

Detailed material and methods are reported in Supplementary Material and Methods section.

## SUPPLEMENTARY MATERIALS FIGURES AND TABLES


















