# Supplementary material for: Luminal breast cancer-specific circular RNAs uncovered by a novel tool for data analysis
Source: Oncotarget. 2018 Feb 19;9(18):14580–96. doi: 10.18632/oncotarget.24522 (PMC5865691; doi:10.18632/oncotarget.24522)
Supplement: Supplementary file 1 [file oncotarget-09-14580-s001.pdf]

Luminal breast cancer-specific circular RNAs uncovered by a novel tool for data analysis

SUPPLEMENTARY MATERIALS

Cell culture

MCF-7, MDA-MB-231, MDA-MB-453 and SK-BR-3 cells were routinely grown in DMEM (Thermo Fisher Scientific, 31053-028); T-47D, T-47D-sfRON and ZR-75-1 cells were grown in RPMI (Thermo Fisher Scientific, 31870-025), HTERT-HME1 and MCF-10A cells were grown in DMEM/F12 (Thermo Fisher Scientific, 21041–025). All media were supplemented with 10% heat-inactivated FBS (Biochrom, S0115-1), 2 mM GlutaMax (ThermoFisher, 35050061), 50 U/ml penicillin and 50 µg/ml streptomycin (Thermo Fisher Scientific, 15140-122), only in DMEM/F12 was added 1x mammary epithelial growth supplement (containing bovine pituitary extract, bovine insulin, hydrocortisone cholera toxin and recombinant human epidermal growth factor). Hormone-Deprived medium (HD) was obtained from phenol red-free DMEM (Thermo Fisher Scientific, 31053-028) supplemented with 5% charcoal-dextran-treated serum and 2 mM GlutaMax (ThermoFisher, 35050061). 17β-estradiol (E2) (Sigma, E2758-1G) was added at a final concentration of 10 nM. Batches of human cell lines were purchased from ATCC. Cell culture was performed at 37°C with 5% CO2.

RNA isolation, RNAse Enrichment and quantitative Real-time PCR (qRT-PCR)

RNA was isolated from MCF-7, MDA-MB-231 SK-BR-3, T-47D, T-47D-sfRON, ZR-75–1, HTERT-HME1, MDA-MB-453 and MCF-10 cells using the Trizol™ reagent (Thermo Fisher Scientific, 15596–026). Nuclear and cytoplasmic RNA fractions were obtained from MCF-7 cell pellets by lysis in 10 mM TRIS pH = 7.8, 140 mM NaCl, 1.5 mM MgCl2, 10 mM EDTA, 0.5% MP40 and 0.3U RNase inhibitor (Thermo Fisher Scientific, AM2694) for 5 min on ice and centrifugation at 3,000× g for 3 min to obtain a cytosolic fraction and nuclear pellet, followed by Trizol™ extraction. Frozen BC tissues (previously collected and stored at − 80°C) were directly homogenized in Trizol™ to extract total RNA. All total RNA samples were subjected to DNase treatment to remove contaminating genomic DNA (DNA-free@ DNA removal kit, Thermo Fisher Scientific, AM1907). First strand cDNA synthesis was performed using the SuperScript™ IV VILO™ reverse transcriptase kit (Thermo Fisher Scientific,11756050). RNase R (Epicentre Biotechnologies) treatment (3U) was performed on total RNA (1 µg) at 20°C for 15 min. qRT-PCR analysis was performed using the SYBR-green method (iTaq Univers SYBR Green, Biorad, 1725124). Real-time PCR primers for human 18S (QT00199367), ERα (QT00044492), GREB1 (QT00080262), GUSBP1 (QT00085204) and Rell1 (QT01662647) RNAs were purchased from Qiagen (QuantiTect@ Primer Assay). Custom expression-primer pairs are reported Supplementary Table 8.

Sanger sequencing

The PCR products were subjected to electrophoresis in 0.8% agarose gel. The fragments were purified and quantified. The Sanger sequencing of PCR products was performed by Bio-Fab research s.r.l. (Rome, Italy) and reported in Supplementary Data 1.

Chromatin immunoprecipitation assay (ChIP)

MCF-7 cells were grown in serum enriched medium (Full medium). ChIP experiments were performed as followed: cells were cross-linked by addition of formaldehyde (formaldehyde 37% stock solution used at 1% final concentration) and incubated 10 minutes at 37ºC. Cross-linking was stopped by addition of glycine solution to a final concentration of 0,125M for 5 minutes on a shaker at room temperature. Cross-linked cells were then washed twice with ice-cold PBS supplemented with complete protease inhibitors cocktail and collect by scraping. Cell pellets were subjected to lysis on ice for 10 min with Lysis Buffer 1 (5 mM Pipes pH 8,85 mM KCl, 0.5% NP40) supplemented with complete protease inhibitors cocktail. Subsequently, nuclei pellets, obtained by a 5 minutes spin cycle at 4ºC (4000 rpm) were exposed once again to 10 minutes lysis in Lysis Buffer 2 (1%SDS, 5 mM EDTA, 50 mM Tris-HCl (pH 8.1) supplemented with complete protease inhibitors cocktail. total extracted chromatin was sonicated to an average size of 200-500 bp. The desired fragments size was checked on 1,2% agarose gel and quantified, in order to use 30 µg of chromatin per IP. Chromatin extracts were diluted with IP-buffer (1% Triton X-100, 2mM EDTA, 20mM Tris-HCl

of pH 8.1, 150 mM NaCl. Supplemented with protease inhibitors before using) and incubated overnight with the specific antibody or IgG at 4°C on a rotating platform. Upon 2 hours of Protein A sepharose beads incubation, samples were washed sequentially for 5 minutes, on a rotating platform with 1 mL of three different Washing Buffer (Wash buffer 1: 0,1% SDS, 1% Triton x100, 2mM EDTA, 20mM Tris-Hcl of Ph 8, 150 mM NaCl; Washing Buffer 2:0,1% SDS, 1% Triton x100, 2mM EDTA, 20mM Tris-Hcl of Ph 8, 500 mM NaCl; washing Buffer 3:0,25 M LiCl, 1% NP40, Na DOC, 1mM EDTA, Tris HCl pH 8) and twice with TE buffer (10mM tris-HCl pH8, 1mM EDTA). After complexes elution at RT with 1% SDS elution buffer (1%SDS, 0.1 M NAHCO3), DNA fragments were de-crosslinked at 65ºC overnight with NaCl 5M and by 1 hour of proteinase K treatment (life technologies, AM2546). DNA purification was achieved with Phenol:Chloroform:IAA (25:24:1) according to the manufacture instructions (Life Technologies, AM9730). qRT-PCR was carried out on ChIP-enriched DNA using SYBR Green Master Mix (iTaq Univers SYBR Green, Biorad, 1725124). ChIP enrichment was normalized on input samples (1% of total chromatin used per IP) and expressed as enrichment of specific binding over the control nonspecific IgG binding. In this assay was used an antibody against H3K36me3 (Active Motif, cat. 61101, lot. 32412003) and Normal Rabbit IgG (Millipore, cat. 12-370). Custom ChIP-primer pairs are reported in Supplementary Table 8.

Small interfering RNA (siRNA)

MCF-7 cells were transfected with siRNAs (20nM final concentration) using Lipofectamine 2000 (Thermo Fisher Scientific, 11668 -019), according to the manufacture protocol. After transfection MCF-7 cells were grown in HD for two days before being harvested. Stealt RNAi from Thermo Fisher Scientific were used to target ERα mRNA (ESR1HSS103376, ESR1HSS103377, ESR1HSS176619). Stealth RNAi™ siRNA Negative Control Med GC was used as a control (siCTR; Thermo Fisher Scientific, 12935–300) Any experiment was carried out at least 48 hours after siRNA transfection to achieve a remarkable efficiency of RNA interference.

Starting RNA-seq datasets and initial prediction of circRNAs in MCF-7

The starting RNA-seq datasets were obtained from libraries generated with the TruSeq stranded library preparation kit (Illumina) using as input material RNA depleted of both poly(A+) and ribosomal RNAs fractions. Each library was analyzed with the DNA 1000 chip (Agilent) using Agilent 2100 Bioanalyzer and quantified using the Qubit DNA HS kit (Lifetechnologies). Pool of 12 libraries (pooled at equimolar concentration) was generated, quantified and run on the HiSeq2000 (Illumina) sequencer in 50 nts paired-end sequencing mode following manufacturer instruction. A total of 12 datasets, with an average depth from 30.7 to 116.1 million paired-end reads, were obtained, composed of triplicates of four MCF-7 culture conditions: i) hormone-deprived (HD) media ii) HD+ 17b-estradiol (6h) iii) medium added of FBS 10% iv) double-stranded RNA complementary to ESR1 mRNA (siRNA) (48h). Raw data are deposited at GSE101410.

CIRI v. 1.2 [1] circRNA prediction analysis was performed aligning RNA-Seq reads with BWA v. 0.6.1 [2] with option *bwasw* and *-T* = 15. Gencode v19 was used as reference transcriptome dataset while hg19 as human reference genome assembly. Read alignment files were used as input for CIRI algorithm applied in default settings with *-P* and *-low* options.

CircRNA prediction with CIRCexplorer v. 1.0.6 was performed following the pipeline proposed in [3]. RNA-Seq reads were aligned using Tophat v. 2.0.0 [4] with options *-bowtie1*, *-a* = 6, *-m* = 2 *-microexon-search -no-novel-juncs*. Unmapped reads were analysed with Tophat-Fusion with options *-fusion-search -keep-fasta-order -bowtie1 -no-coverage-search.* The CIRCexplorer algorithm was used in default settings on the output of Tophat-Fusion.

CircRNA prediction analysis with find_circ v. 1.2 [5] as performed by aligning reads using Bowtie v. 2.0 [6] with options *-very-sensitive -phred33 -mm -D* = 20 *-score-min* = C,-15,0. Unmapped reads were used as input for the find_circ pipeline following the procedure proposed in [5].

For each analysis, the number of BS reads reported by each algorithm was normalized using DESeq2 v.1.14.1 R package [7].

On each set of circRNA predicted by the three algorithms, the circRNAs predicted in at least two out of the three biological replicates in each culture condition and associated with an average number of BS supporting read > 2 were selected. Using this threshold 3,271, 1,811, and 2,797 circRNAs were predicted with CIRI, find_circ and CircExplorer, respectively.

The circRNA genomic coordinates were used to compare the predictions of the three algorithms and the annotations of CircBase [8] (release Dec 15th, 2015) and circRNAdb v. 1.0.0 [9]. The comparison was performed by considering the circRNAs genomic coordinates.

Representation of the genomic regions involved in the BS events was performed using the Washu EpiGenome Browser v42 [10].

CIRI algorithm was applied with the same settings to predict circRNA from public RNA-Seq experiments from ENCODE projects performed using total RNA (GSM2072571, GSM2072572), poly(A)+ (GSM767851), and poly(A)- (GSM765388) RNA selection protocols. CIRI was applied with same settings used on our RNA-Seq datasets and only circRNAs identified in both the biological replicates of the experiments were considered for the analysis.

CircHunter is a new tool designed for the post-discovery analysis of circRNA predictions. CircHunter is composed by three modules (i) circRNA classification,

(ii) BS sequence reconstruction, and (iii) BS sequence quantification in deep sequencing datasets.

In the circRNA classification module the algorithm considers the annotation from a reference transcriptome, which in our analysis was Ensembl v85. Initially, genomic coordinates of each Ensembl exon are overlapped against circRNA genomic coordinates using bedtools [11] *intersect* function. Then, the genomic coordinates of each exon annotation are tested for the overlap against circRNA BS site position. Each overlap is classified based on the number and the position of the BS within the transcript annotations. Each circRNA/transcript overlap is classified to five possible criteria (Supplementary Figure 1B):

multiexonic, when two exons are mapped to each splice site of the circRNA;

monoexonic, when a single exon spans the entire region involved in the circularization;

putative exonic, when there is no precise match between the circRNA BS sites and the exon boundaries but BS sites is mapped within exon genomic coordinates;

intronic; when at least one intron is mapped to a circRNA BS site;

intergenic; when at least one circRNA BS sites exceeds the boundaries of the associated gene.

This analysis provides a transcript-level classification of circRNA-overlapping transcript. Then, a single circRNA can be associated with multiple classifications when overlapped on multiple transcripts. To obtain the univocal classification of each circRNA, the main isoform of the circRNA host genes is considered by selecting the Ensembl transcript identified with the suffix “001”. If none main isoform is overlapped with a circRNA, the other isoforms are evaluated following the order provided by Ensembl.

The circRNA nomenclature applied in this work was based on the isoform considered for the univocal classification. Specifically, each circRNA name was composed by the prefix “Circ” followed by the host gene symbol and ended with the rank of 5’ and 3’ exons involved in the circularization. Intergenic circRNAs were named based reporting their genomic coordinates while intronic circRNAs were distinct by the “I” suffix. The univocal classification was considered in the analysis of the number and rank of the exons involved in the BS event.

The circRNA BS sequence reconstruction module applied a python script which select two set of genomic coordinates starting from the BS sites and involving a portion of BS exon selected by the user (default length is 35 bp). An R script is then applied to convert the genomic coordinates in R *GRanges* objects. Then, the BS sequence is reconstructed using the function *getSeq* and *xscat*. These functions were applied respectively to extract and to concatenate properly the two sequences composing the BS junction.

The BS sequence quantification in deep sequencing datasets module is performed by *HashCirc*. *HashCirc* is organized on three steps: in the first and second steps an alignment-free prediction method is exploited to identify the set of putative sequencing reads mapped on the sequences of interest; while in the third step the selected putative reads are aligned against the sequences of interest (i.e. circRNA BS junction sequences) to generate the corresponding *counting table* (i.e. the counting of the number of reads aligned with each sequence).

Step 1: Significant k-mer generation. In this step, the entire set of sequences is scanned and a set of substrings with length *k*, namely k-mers, is generated using a *sliding window* approach.

For instance, given a string *ATCCCGTC* the following k-mers with length three are generated: *ATC, TCC, CCC, CCG, CGT* and *GTC.*

Then, a hashing is exploited to build the function *isPresent*: $\{A,C,G,T\}_k \rightarrow [0,1]$ which, for each k-mer, returns one if it appears in any sequence otherwise 0.

A k-mer α is considered significant and therefore selected if *isPresent(*α*)*=1. These selected k-mers will be used to identify the putative reads in the next step.

Step 2: read selection. In this step hashing is still used to build function *check*: $\{A,C,G,T\}_k \rightarrow \{0,1\}$, which for each k-mer returns *1* if it is a selected k-mer otherwise *0*. Then, the function *check* is applied on all the k-mers of a read so that a read is selected as putative one if it contains more than *N* k-mers for which *check* function returns 1.

Step 3: read counting. The derived set of putative reads are hence aligned w.r.t the sequences. For each read, its best alignment with respect to all the sequences is identified and used to generate the sequence counting table. In the CircHunter tool suite, the *HashCirc module*, is composed of two C++ applications for each step of the data processing:

The first step takes as input a set of sample reads, the set of sequences and the threshold *N,* and returns the corresponding set of putative reads which contain at least N k-mer shared with the set of sequences. The k-mers generated by the sequences are stored in RAM exploring an ad-hoc C++ hash table class implementation to optimize the trade-off between the memory utilization and the execution time.

The second step takes as input the set of putative reads for each sample, it counts the frequency of a set of reference sequences (i.e. pre-defined BS sequences). For this step, the Smith-Waterman algorithm provided by SIMD Smith-Waterman C++ library is used.

To perform the *HashCirc* analysis, sequences of 70 bp representing the hypothetical circRNA BS junctions were extracted from the circRNA predictions using *CircHunter*. Two set of genomic coordinates spanning +35/-35 bp from the junction point respectively were generated using this algorithm and circRNAs shorter than 70 bp were splitted in two halves used for the junction

reconstruction. The efficiency of *HashCirc* in circRNA quantification was evaluated by Pearson correlation analysis between the number of reads counted by *HashCirc* with the reads reported by CIRI. ENCODE MCF-7 RNA-Seq experiments were also considered for analysis of the algorithm sensitivity in circRNA detection. For this analysis data from Poly(A)+ (GSM767851), Poly(A)- (GSM765388), and total RNA-Seq datasets (GSM2072571, GSM2072572) were analyzed by setting the k-mer length (*k*) to 26, the minimum number of matched k-mer (*N*) to 21, and the minimum number of matches (*M*) equal 40. A set of 75-bp simulated paired-end reads from [12] was considered as circRNA negative set since it was generated from linear mRNA annotations. A ratio between the reads counted by HashCirc in the PolyA- and the simulated read datasets was computed to evaluate the rate of false positive count. The results of these testing analyses are reported in Supplementary Data 1.

To make easy the use of this new pipeline a Graphical User Interface (GUI) based on Java Swing Class was developed too. Moreover, the pipeline was integrated into a Docker container to facilitate its distribution and installation. It can be downloaded at https://github.com/carlo-deintinis/circhunter

Public RNA-seq analysis

The public RNA-Seq experiments were analyzed by reads alignment against Gencode v19 annotations and Hg19 genome using Tophat v. 2.0.0 in default settings. Read count was performed using the featureCount algorithm v.1.5.0-p1 [13] and read count table normalized using DESeq2 v.1.14.1 R package. Normalized read counts were then converted in Fragment Per Kilobase exon per Million mapped reads (FPKM) considering the length of the longest isoform and the million number of read counted by featureCount.

circRNAs host genes genomic characterization

The genomic features of 1,761 circRNA host genes were compared against selected control gene set (*Control gene set*) and 1,000 random gene sets (*Random gene set*). Specifically, the Control gene set was defined by selecting genes lacking circRNA predictions considering the union between circBase, circRNADb annotations and circRNAs predicted in this study. To select control genes expressed in MCF-7, a public Poly(A)+ MCF-7 RNA-Seq experiment performed in full medium (GSE48213, [14]) was re-analyzed and genes associated with a FPKM greater than 1 was considered as expressed. Using these two criteria 5,583 genes were selected as control. The lack of significant difference between host and control genes expression level was confirmed by Wilcoxon Rank-Sum test (*p*-value = 0.7957). A Random gene set was composed by 1,000 sets of 1,761 genes randomly selected from Gencode v19 annotations.

Function enrichment analysis of circRNA host gene and the Control gene set was performed using Enrichr web tools [15]. The comparison of gene/transcript length, the number of exons and isoforms between circRNA host genes and control set was performed using the Ensembl annotations. Data of the main gene isoforms (reported with suffix "001" by Ensembl) were used in this analysis.

Analysis of candidate circRNA intronic retention events was performed by considering reads paired with each BS spanning read. The genomic coordinates of these reads were retrieved from BWA alignment outputs using BS read identifiers provided by CIRI. Then, the reads genomic coordinates were mapped against Ensembl exon genomic coordinates. Only perfectly matched reads were considered for this analysis.

Analysis of Alu element annotated in the intronic regions flanking the circRNA BS exons was performed by considering the Alu annotations downloaded from UCSC using the RepeatMasker track and by overlapping their coordinates with an intronic region of 500 bp flanking the circRNA BS junctions as previously performed [16].

Analysis of the epigenetic status of circRNA genomic regions

The epigenetic status at the genomic region involved in the BS events was evaluated by overlapping the BS genomic coordinates with 15 chromatin states defined for the MCF-7 epigenome in full medium culture condition [17]. Data of ChIP-Seq experiment against H3K27ac (GSM1383859), H3K36me3 (GSM970217), H3K4me3 (GSM1383862) and RNAPII (GSM1276019) were also analyzed to measure the ChIP-Seq genomic signal profile around 5' or 3' BS sites. Specifically, ChIP-Seq reads were aligned against hg19 human genome using Bowtie2 in default settings. The genomic signal profile was then computed using seqMINER algorithm v1.3 [18] considering a genomic region of +/- 1 kbp centered on the BS sites. The genomic signal normalization was then normalized using *NormChIP* algorithm [17]. The number of H3K36me3 read covering the first five exons of circRNA host genes or the control set were counted using the *coverageBed* function of bedtools.

Ago-HITS CLIP data analysis and MRE prediction

Raw Ago-HITS-CLIP data and Ago-HITS-CLIP peaks were retrieved from GSE57855. The raw sequencing reads were aligned using Bowtie2 algorithm in default settings and reads aligned within a genomic region of +/- 1 kbp centered on circRNA BS sites were counted. As control the corresponding splicing sites of exon 2 and exon 3 from genes were analyzed.

MRE prediction was performed on the reconstructed sequence of exonic circRNAs composed of one two, or three exons. MRE prediction was performed using the Miranda algorithm [19] in default settings and considering mirBase v20 annotations. Overlap with circRNA exons and Ago-HITS-CLIP was performed using coverageBed

function of bedtools. To analyze miRNAs expressed in MCF-7 cells, processed data from small RNA-Seq experiments from GSE78168 were considered. Only miRNAs associated with an average Read Per Million Mapped reads (RPPM) > than 100 in E2 untreated cells were considered.

HashCirc was applied on Ago-HITS-CLIP to identify Ago-RNA binding generated by BS events. Given the smaller length of Ago-HITS-CLIP reads the algorithm was applied with settings k = 21, N = 17, and M = 30. Read count normalization was performed using DESeq2 algorithm. MRE prediction was performed on the reconstructed BS sequence of circRNA subset associated with an Ago-HITS-CLIP signal as defined by HashCirc analysis. Different control sets were defined for this analysis: 1) 100 sets of 3,271 sequences generated by randomly permuting the 3,271 CM7 BS sequences; 2) 100 sets of sequences generated by randomly selecting and permuting 127 CM7 BS sequences; 3) 100 sets generated by randomly permuting the 127 BS sequences overlapped with the Ago-HITS-CLIP datasets; 4) 100 set of sequences generated by randomly shuffling the 3,271 CM7 BS sequences.

Considering the CM7 BS sequences associated with more than six averaged Ago-HITS-CLIP reads, the overlap with the splicing junction of the linear mRNA sequences was evaluated. The overlap was performed by selecting the splicing junction sequence in between of the exons involved in the circularization and also by considering the junction formed between the circularizing exons and the upstream and downstream exons. Direct sequence alignment between Ago-HITS-CLIP reads and the splicing junctions was performed using Bowtie2 algorithm with local option. The read coverage was computed using Samtools pileup function.

CircRNA expression analysis in public total RNA-Seq experiments

The analysis of circRNA expression in five BC cell lines and one non-tumorigenic cell line was performed considering total RNA-Seq experiments from GSE52643. The reads aligned against the circRNA BS junction were counted using *HashCirc* with settings k = 21, N = 17, and M = 30. For the analysis of circRNA expression in primary tumor tissues (GSE52194), total RNA-Seq data of 20 BC tumors and 3 NBO were analyzed with *HashCirc* with settings k = 22, N = 18, and M = 33. The different settings were selected based on the different length of the RNA-Seq reads analyzed.
The read count normalization and the DE analysis were performed using DESeq2. The DE analysis on cell lines data was performed between ER+ (MCF-7, T47-D, ZR-75.1) and ER- (BT-474, MDA-MB-231, MCF-10A) cell lines. The DE analysis of circRNA expression in tumor tissues was performed by comparing ER+ tumors against NBO, HER2+ amplified tumors, or Triple Negative (TN) tumors. A circRNA was considered significantly DE if associated with a *p*-value lower than 0.05.

Radar plot representation of candidate circRNAs expression in tumor tissues was performed using the *fmsb* R package.

REFERENCES

1. Gao Y, Wang J, Zhao F. CIRI: an efficient and unbiased algorithm for de novo circular RNA identification. Genome Biol. 2015; 16:4.
2. Li H, Durbin R. Fast and accurate short read alignment with Burrows-Wheeler transform. Bioinformatics. 2009; 25: 1754–60.
3. Zhang X-O, Wang H-B, Zhang Y, Lu X, Chen L-L, Yang L. Complementary Sequence-Mediated Exon Circularization. Cell. Elsevier Inc.; 2014; 159:134–47.
4. Kim D, Pertea G, Trapnell C, Pimentel H, Kelley R, Salzberg SL. TopHat2: accurate alignment of transcriptomes in the presence of insertions, deletions and gene fusions. Genome Biol. 2013; 14:R36.
5. Memczak S, Jens M, Elefsinioti A, Torti F, Krueger J, Rybak A, Maier L, Mackowiak SD, Gregersen LH, Munschauer M, Loewer A, Ziebold U, Landthaler M, et al. Circular RNAs are a large class of animal RNAs with regulatory potency. Nature. 2013; 495:333–8.
6. Langmead B, Salzberg SL. Fast gapped-read alignment with Bowtie 2. Nat Methods. 2012; 9:357–9.
7. Love MI, Huber W, Anders S. Moderated estimation of fold change and dispersion for RNA-seq data with DESeq2. Genome Biol. 2014; 15: 550.
8. Glažar P, Papavasileiou P, Rajewsky N. circBase: a database for circular RNAs. RNA. 2014; 20:1666–70.
9. Chen X, Han P, Zhou T, Guo X, Song X, Li Y. circRNADb: A comprehensive database for human circular RNAs with protein-coding annotations. Sci Rep. 2016; 6:34985.
10. Zhou X, Wang T. Using the Wash U Epigenome Browser to examine genome-wide sequencing data. Curr Protoc Bioinforma. 2012; Chapter 10:Unit10.10.
11. Quinlan AR, Hall IM. BEDTools: a flexible suite of utilities for comparing genomic features. Bioinformatics. 2010; 26:841–2.
12. Carrara M, Beccuti M, Lazzarato F, Cavallo F, Cordero F, Donatelli S, Calogero RA. State-of-the-art fusion-finder algorithms sensitivity and specificity. Biomed Res Int. 2013; 2013:340620.
13. Liao Y, Smyth GK, Shi W. featureCounts: an efficient general purpose program for assigning sequence reads to genomic features. Bioinformatics. 2014; 30:923–30.
14. Daemen A, Griffith OL, Heiser LM, Wang NJ, Enache OM, Sanborn Z, Pepin F, Durinck S, Korkola JE, Griffith M, Hur JS, Huh N, Chung J, et al. Modeling precision treatment of breast cancer. Genome Biol. 2013; 14:R110.
15. Kuleshov M V, Jones MR, Rouillard AD, Fernandez NF, Duan Q, Wang Z, Koplev S, Jenkins SL, Jagodnik KM, Lachmann A, McDermott MG, Monteiro CD, Gundersen GW, et al. Enrichr: a comprehensive gene set enrichment

analysis web server 2016 update. Nucleic Acids Res. 2016; 44:W90–7.

16. Jeck WR, Sorrentino JA, Wang K, Slevin MK, Burd CE, Liu J, Marzluff WF, Sharpless NE. Circular RNAs are abundant, conserved, and associated with ALU repeats. RNA. 2013; 19: 141–57.

17. Ferrero G, Miano V, Beccuti M, Balbo G, De Bortoli M, Cordero F. Dissecting the genomic activity of a transcriptional regulator by the integrative analysis of omics data. Sci Rep. 2017; 7:8564.

18. Ye T, Krebs AR, Choukrallah MA, Keime C, Plewniak F, Davidson I, Tora L. seqMINER: An integrated ChIP-seq data interpretation platform. Nucleic Acids Res. 2011; 39:e35.

19. Enright AJ, John B, Gaul U, Tuschl T, Sander C, Marks DS. MicroRNA targets in Drosophila. Genome Biol. 2003; 5:R1.

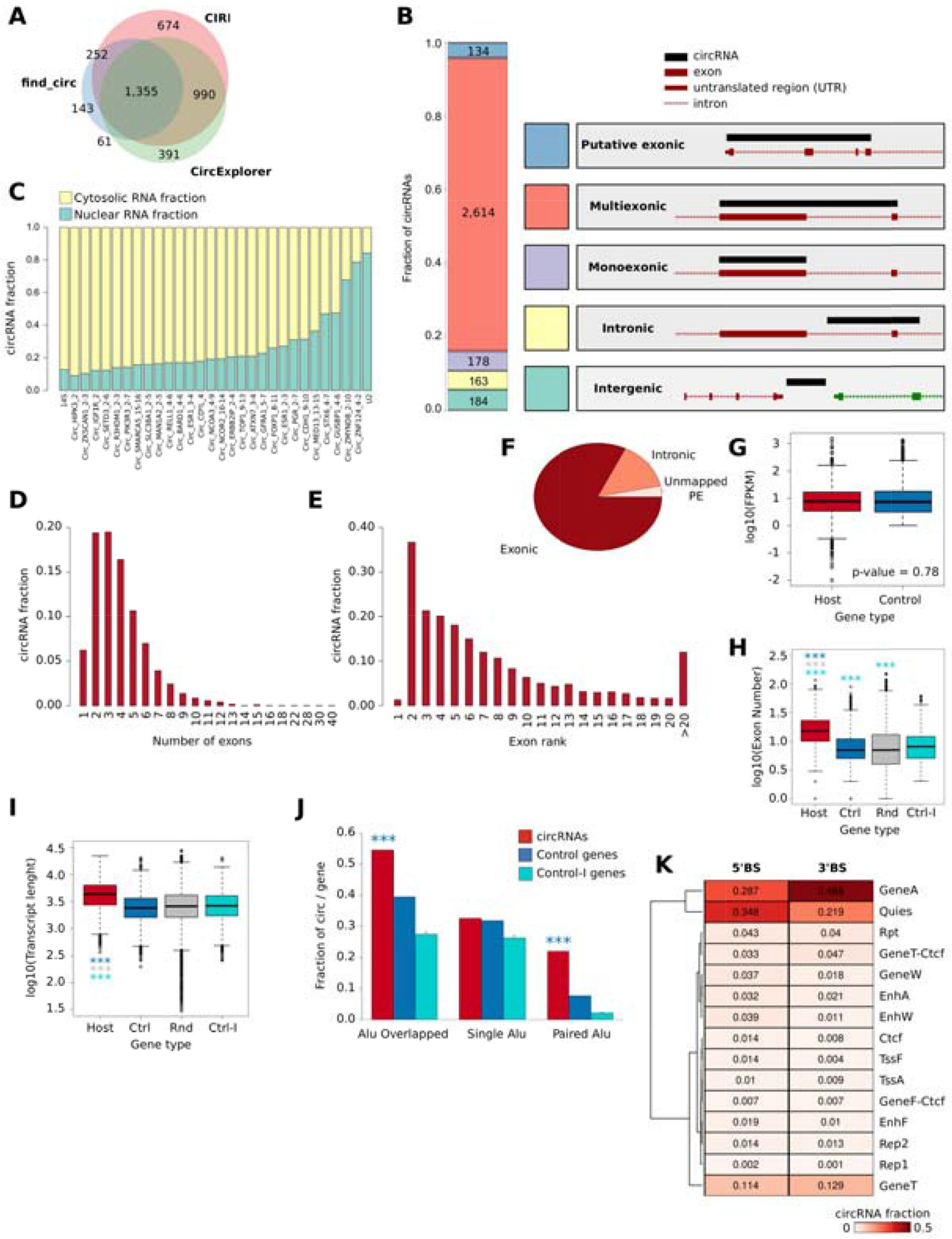

Supplementary Figure 1: (**A)** Venn diagram shows the overlap between circRNAs predicted by three different algorithms, CIRI (pink), find_circ (blue), and CircExplorer (green). (**B)** Bar plot shows the circRNAs classification based on their genomic position. (**C)** Bar plot represent the cellular distribution of a 28 circRNA as measured by qRT-PCR on nuclear or cytosolic MCF-7 RNA fraction. 14S and U2 are used as a cytoplasmic and nuclear controls respectively. The experiment was performed in triplicate. (**D)** Bar plot shows the fraction of circRNA characterized by a specific number of exons involved in circularization. (**E)** Bar plot represents the position of the exons forming BS events. (**F)**Pie chart shows the fraction of circRNAs in which the BS-paired reads was mapped inside an exon (dark red) in an intronic sequence (light red) or unmapped (pink). (**G)** Box plot shows the level of circRNA host (red) and control genes (blue) expression in MCF-7. Expression levels is reported as log10 Fragment Per Kilobase exon per Million mapped reads (FPKM). *P*-value by Wilcoxon Rank-Sum Test. (**H)** Box plot representing the number of exons on host genes (red), control genes (Ctrl, blue), random genes (Rnd, grey), and control genes paired with host genes by the first intron length (Ctrl-I, cyan). *P*-value by Wilcoxon Rank-Sum test. *** = *p*-value < 0.001. (**I)** Box plot represents the length of the longest transcript annotated for the host genes (red), control genes (Ctrl, blue), random genes (Rnd, grey), and control genes paired with host genes by the first intron length (Ctrl-I, cyan). *P*-value by Wilcoxon Rank-Sum test. *** = *p*-value < 0.001. **(J**) Bar plot reports the fraction of host genes (red), control genes (Ctrl, blue) and control genes paired with host genes by the first intron length (Ctrl-I, cyan) associated with an Alu element annotated within introns flanking the circularizing exons. The result is reported also for the analysis performed considering only the single or paired Alu elements. *P*-value by Chi-square test. *** = *p*-value < 0.001. (**K)** Heat map represents the fraction of circRNA whose BS sites is overlapped with a specific chromatin states as defined in [17].

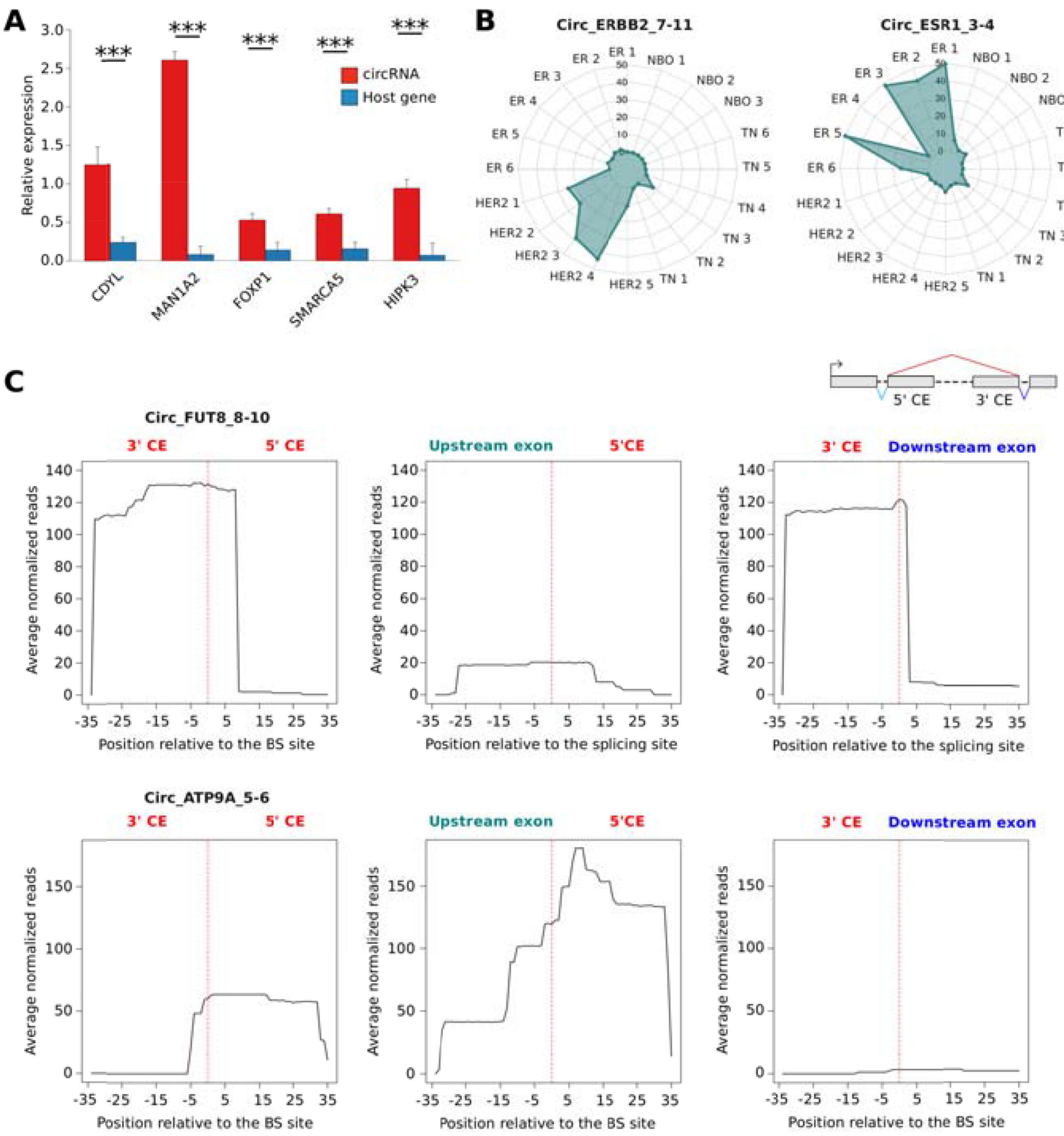

Supplementary Figure 2: (**A**) Bar plot shows the relative expression of circRNAs (red) and their corresponding host genes (blue) measured by qRT-PCR in MCF-7. Bars represent standard deviation from three independent biological replicates. *P*-value from Student *t* test. *** = *p*-value < 0.001. (**B)** Radar plot represents the number of BS reads counted by *HashCirc* in 20 total RNA-Seq datasets from primary tumors specimens for Circ_ERBB2_7-11 and Circ_ESR1_3-4 circRNAs. (**C**) Coverage plot representing the number of AGO-HITS CLIP reads covering the sequence of the BS (right), the upstream (center) and the downstream (left) junction by direct sequence to sequence alignment.

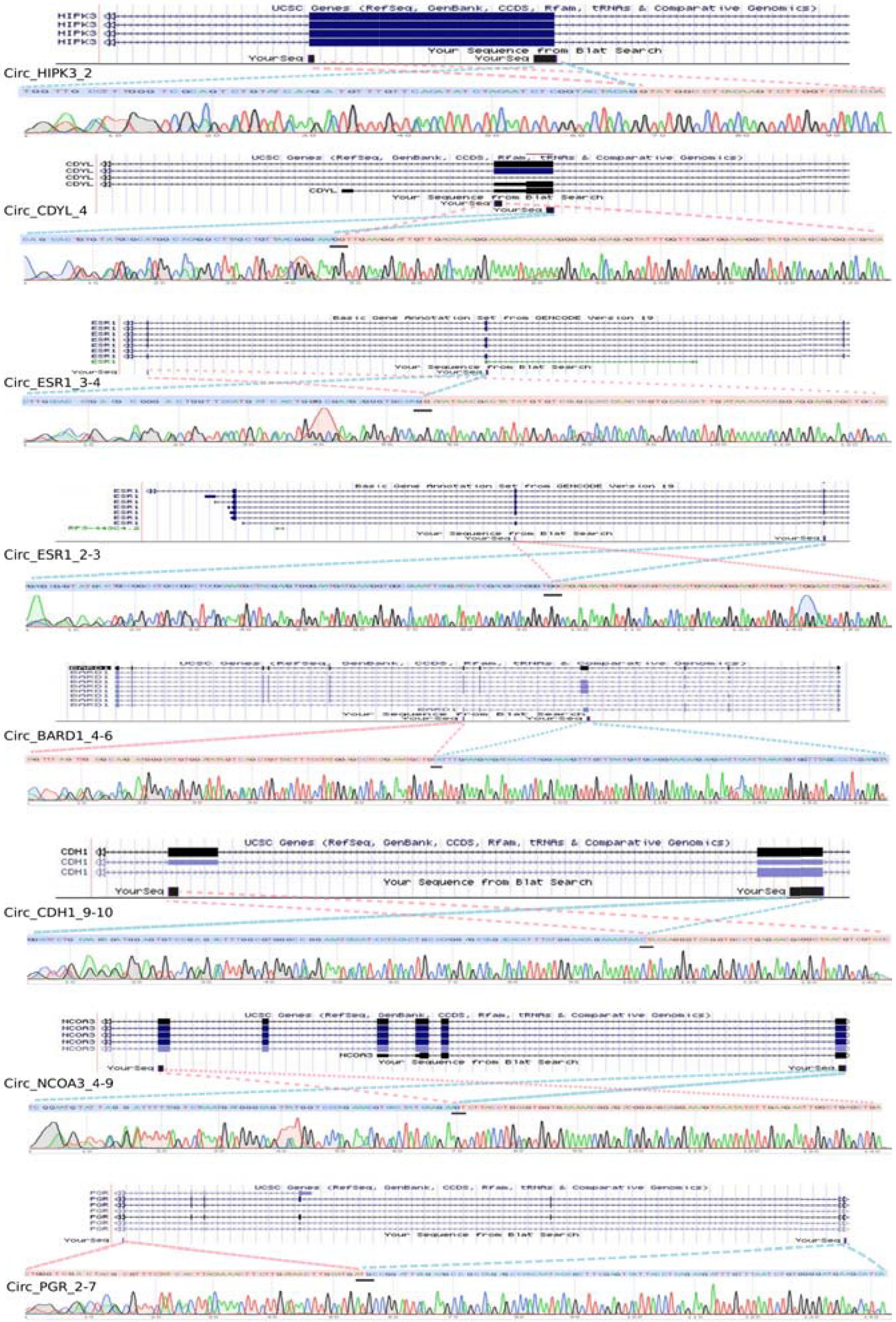

Supplementary Data 1: Electropherogram plot reporting the sequence of eight CM7 circRNA BS junction sequenced by Sanger sequencing technique.

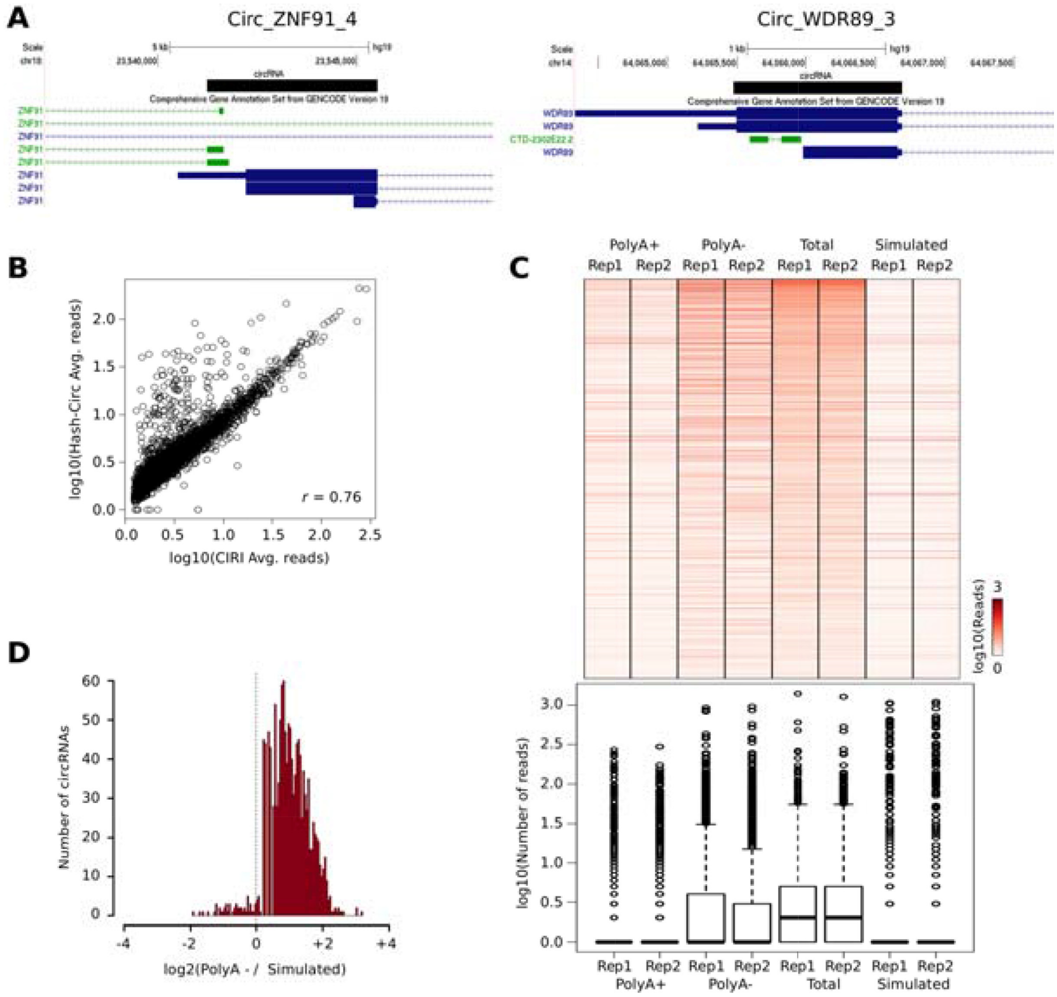

Supplementary Data 2: (**A**) Genome Browser representation of the genomic localization of two circRNAs classified as putative exon by *CircHunter*. (**B)** Scatter plot represents the log10 average number of BS supporting reads predicted by CIRI algorithm (x axis) and the log10 average number of BS supporting reads counted by *HashCirc* module of *CircHunter* (Y axis). **(C)** Heat map represents the number of BS supporting reads counted by *HashCirc* module of *CircHunter* in RNA-seq experiments performed in MCF-7 by ENCODE using three different RNA purification methods (poly(A)+, Poly(A)- and total RNA) and a negative set of simulated 75 nucleotide reads generated using Ensembl annotations (lacking of back-splicing junctions*)*. The read count distribution is reported as box plot at the bottom. (**D)** Histogram representing the number of circRNAs characterized by a specific log2 ratio computed between polyA- and simulated RNA-seq reads counted by *HashCirc*.

Supplementary Table 1: Summary of CM7 information, number of back-splicing reads and genomic characterization. See_Supplementary_Table 1

Supplementary Table 2: Summary of circRNA host gene ontologies. See_Supplementary_Table 2

Supplementary Table 3: Enrichment analysis of H3K36me3 on CM7 exons. See_Supplementary_Table 3

Supplementary Table 4: Result of the differential expression analysis of CM7 expression in different breast cancer cell lines. See_Supplementary_Table 4

Supplementary Table 5: Result of the differential expression analysis of CM7 in expression different breast tumour tissues. See_Supplementary_Table 5

Supplementary Table 6: Summary of tumour tissues clinical data. See_Supplementary_Table 6

Supplementary Table 7: List of AGO overlap with CM7 and analysis of miRNA regulatory elements. See_Supplementary_Table 7

Supplementary Table 8: List of expression and ChIP primer sequences. See_Supplementary_Table 8
